# Global distribution and dynamics of muddy coasts

**DOI:** 10.1038/s41467-023-43819-6

**Published:** 2023-12-13

**Authors:** Romy Hulskamp, Arjen Luijendijk, Bas van Maren, Antonio Moreno-Rodenas, Floris Calkoen, Etiënne Kras, Stef Lhermitte, Stefan Aarninkhof

**Affiliations:** 1 Deltares, Boussinesqweg 1, 2629 HV Delft, The Netherlands; 2 Faculty of Civil Engineering and Geosciences, Delft University of Technology, Delft, The Netherlands; 3 State Key Lab of Estuarine and Coastal Research, East China Normal University, Shanghai, China; 4 Department of Earth and Environmental Sciences, KU Leuven, Leuven, Belgium

**Keywords:** Geomorphology, Civil engineering, Sedimentology, Geomorphology

## Abstract

Muddy coasts provide ecological habitats, supply food and form a natural coastal defence. Relative sea level rise, changing wave energy and human interventions will increase the pressure on muddy coastal zones. For sustainable coastal management it is key to obtain information on the geomorphology of and historical changes along muddy areas. So far, little is known about the distribution and behaviour of muddy coasts at a global scale. In this study we present a global scale assessment of the occurrence of muddy coasts and rates of coastline change therein. We combine publicly available satellite imagery and coastal geospatial datasets, to train an automated classification method to identify muddy coasts. We find that 14% of the world’s ice-free coastline is muddy, of which 60% is located in the tropics. Furthermore, the majority of the world’s muddy coasts are eroding at rates exceeding 1 m/yr over the last three decades.

## Abstract (web summary)

14% of the world’s coastlines are muddy and the majority of them are eroding at rates exceeding 1 m per year over the last three decades, according to an automated classification method that identifies global coastlines.

## Introduction

Coastal zones are vulnerable to flooding resulting from storms, or coastline erosion resulting from a relative sediment deficit; the latter caused by trapping of sediment in upstream reservoirs^1,2^ and subsidence^3^. On the other hand, globally averaged deltas still experience net land growth over the past 30 years, largely due to larger sediment loads resulting from deforestation^4^. However, in the next decades, relative rise in sea level provides an increasingly large challenge for the maintenance of coastlines, because eustatic sea level rise is increasing^5,6^ and many deltas are sinking due to groundwater extraction^3^. On top of relative sea level rise and loss of sediment supply, many coastal areas will also experience increasing mean and extreme coastal wave energy^7^ in the coming decades. Sustainable management of these coastal areas therefore requires a thorough understanding of the past and future coastline trends. One way of advancing our understanding of coastline dynamics is by evaluating coastline response on a global scale, providing a methodology to relate coastline dynamics to global changes. Such studies exist for sand-dominated coastlines^8,9^ and for deltas on a more aggregated level^4^. However, despite the global abundance of mud-dominated coastlines, a global inventory of mud coast prevalence and dynamics does not yet exist.

A muddy coast is here defined as a sedimentary-morphodynamic type characterised primarily by fine-grained sedimentary deposits (silts and clays) forming flat surfaces often (but not exclusively) associated with broad tidal flats^10^. These coastal environments are usually associated with rivers carrying a large sediment load, such as the Amazon River, the Mississippi River and the large Asian rivers draining the Himalayas (notably the Yellow, Yangtze, Red, Mekong, Ayerwaddy, Ganges-Brahmaputra and Indus). The existence of a muddy coast requires a supply of mud exceeding its alongshore and cross-shore dispersal rate. On their landward side most mud coasts are flanked by salt marshes (in temperate regions) or mangroves (in the tropics). These ecosystems offer ecological habitats, provide food, and form a natural coastal defence against storms^11^. Mud coasts, especially in combination with salt marshes or mangroves, therefore provide important ecosystem services. To better understand the global distribution and the dynamics of mud coasts, we developed a methodology to detect mud-dominated coasts and its dynamics.

Muddy coastlines are more difficult to detect compared to sandy coastlines because the contrast between land and (muddy) water is less pronounced. Therefore we develop a hybrid machine learning method combining classified multispectral satellite imagery with global coastal geophysical datasets. Through supervised machine learning this methodology classifies coastlines into five coastal geomorphological types: sandy coasts, muddy coasts, rocky coasts, vegetated coasts and other. By sampling large amounts of training and validation data at both pixel (*n* = 3240) and transect level (*n* = 1868), we were able to develop an algorithm that identifies muddy coasts with a high accuracy.

## Results

### Global occurrence of muddy coasts

**Fig. 1:**
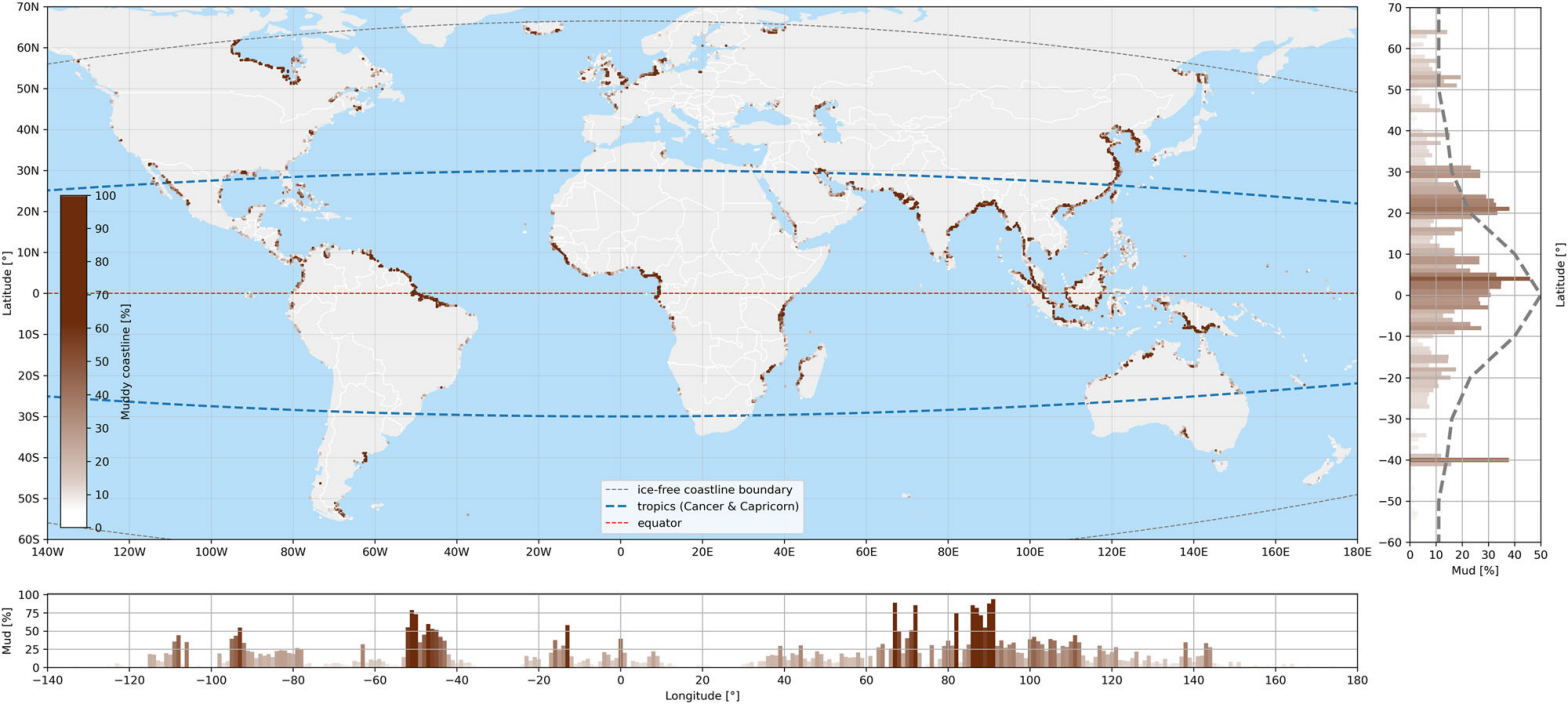
Global distribution of muddy coastlines derived from the hybrid classification model, with colours denoting the local percentage of mud (darker brown implying more mud). The subplot on the right averages the relative occurrence of muddy coastlines per latitudinal degree, with the dashed line representing the distribution of muddy coastlines reported by Hayes^18^. The lower subplot shows the relative occurrence of muddy coastlines per longitudinal degree. The curved grey dashed lines in the main plot indicate the boundaries of the ice-free coastlines considered in this analysis. The curved blue dashed lines represent the Tropic of Cancer and the Tropic of Capricorn, the red dashed line indicates the equator.

The hybrid coastal transect classification model reaches an accuracy of 76.0% when classifying all five geomorphological types; the addition of geospatial data to the multispectral classification improves the classification accuracy with 11.2%. Muddy coasts are more accurately classified compared to the average coastal type, with an accuracy of 86.5%. The hybrid classifier clearly reveals several clusters of muddy coastlines corresponding to well known mud-dominated coastlines (Fig. 1). In the American continent, the most prominent mud coastlines are the Amazon-influenced coastlines^12^ and the Hudson Bay^13^. The most extensive muddy coastline in Europe is the Wadden Sea^14^, whereas the mud-dominated coastlines of West Africa^15^ also clearly emerge. However, the most prominent mud-dominated coasts are observed in Asia (particularly the Indian subcontinent, China and Indonesia). This can be explained by the fact that this region receives about 70% of the global suspended sediment load because its sediment yield is much greater than other drainage basins^16^.

Global integration of these findings reveals that 14% of the world’s ice-free coastline is muddy, of which 60% is located between 25^*◦*^N and 25^*◦*^S. The latter is in accordance with Flemming^17^ who estimated that muddy coasts neighbouring mangrove systems in this region cover an area of about 75%. The latitudinal distribution is consistent with the distribution of Hayes^18^ and reflects the large suspended load carried by tropical rivers^16^. The longitudinal distribution reflects the large suspended load carried by rivers draining the Himalayan and Southeast Asian rivers, as well as the Amazon river.

Our analyses estimate a total of 91,400 km (95% CI 82,600−101,500 km) of muddy coastlines of which 39,700 km (95% CI 39,380 − 40,130 km) fall within the muddy areas previously reported in a comprehensive literature survey by Flemming^17^.

### Dynamics of muddy coasts vs. sandy coasts

Globally, sandy coastlines are relatively stable, with approximately as much eroding beaches as accreting beaches^8^. Although the global dynamics of undifferentiated coastlines (without distinguishing between the coastline type) have been investigated^4,9^, a methodology to differentiate between the dynamics of muddy coasts and sandy coasts does not yet exist. This is important, because the processes driving their long-term stability, the role of human interventions as well as climate change, is very different for muddy coastlines than for sandy coastlines. Many river deltas are mud-dominated and relatively flat, and therefore constitute vast low-lying areas which are sensitive to the delicate balance between sea level rise, land subsidence and sedimentation^19^. To derive the historical changes of muddy coastlines, we adopt the method of Luijendijk et al.^8^ for the period 1984–2016. This methodology was developed and validated for sandy beaches, requiring slight adaptations for application to mud-dominated coastlines (see Supplementary Material (SM)). After filtering the muddy coastline transects for a minimum number of historic data points, temporal coverage, potential ice coverage and outliers, 97.7% of the mud coasts remain suitable for further analysis.

The analysis of coastline dynamics reveals that 40% of the world’s muddy coasts are stable (absolute long-term change rates of <1 m/year over the period 1984–2016); 31% are persistently eroding, while 29% are accreting (Table 1). About 7.8% of the muddy coasts exceed erosion rates of more than 10 m/year, while 8.8% are accreting at rates of more than 10 m/year. On a global scale, the world’s muddy coasts have accreted 0.18 m/year on average over the three decades of the study period, i.e. a total gain of 579 km^2^ over this period. Muddy coastlines are generally more dynamic than sandy coasts, reflected in much more (very) extreme accretion and erosion. Rapid accretion rates can be explained by the the dominance of fine-grained suspended load in the world’s major river systems^16^; rapid erosion rates by the low gradient of mud-dominated coastal environments (resulting in large horizontal erosion rates for a given vertical erosion rate).

**Table 1:** Chronic coastline dynamics classification scheme

Dynamic class	Rate	Sandy coasts (%)	Muddy coasts (%)
Very extreme accretion	*>*10 m/yr	1.6	8.8
Extreme accretion	5 to 10 m/yr	1.8	5.4
Severe accretion	3 to 5 m/yr	2.3	4.3
Intense accretion	1 to 3 m/yr	10.1	10.7
Stable	−1 to 1 m/yr	69.7	40.3
Intense erosion	−1 to −3 m/yr	9.8	12.2
Severe erosion	−3 to −5 m/yr	1.9	4.8
Extreme erosion	−5 to −10 m/yr	1.5	5.7
Very extreme erosion	*<*−10 m/yr	1.2	7.8

**Fig. 2:**
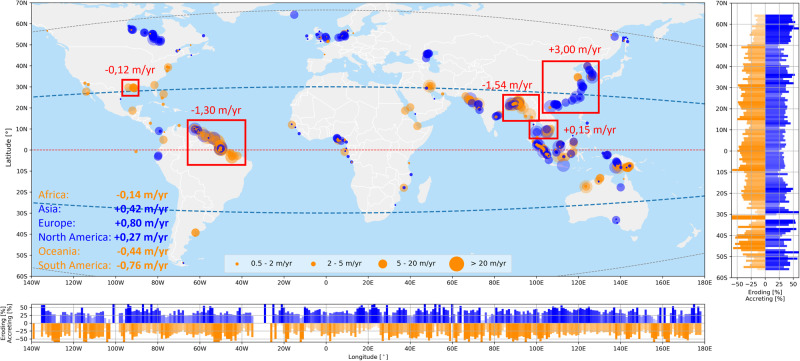
Global hot spots of muddy coast erosion and accretion; the orange (blue) circles indicate erosion (accretion) for the four relevant coastline dynamic classifications (see legend). The bar plots to the right and at the bottom present the relative occurrence of eroding (accreting) muddy coastlines per degree latitude and longitude, respectively.

Mapping of muddy areas experiencing rapid accretion and erosion reveals several erosion and sedimentation clusters (Fig. 2). Coastline changes may be the result of changes in the sediment load^1,2,4^ (i.e. expansion of a high natural sediment load or a more recent increase resulting from deforestation^4^) and subsidence^3^.

Coastline change is most pronounced in South, Southeast and East Asia, representing 57% of all land globally gained by river deltas but also where 61% of all deltaic land loss occurs^4^. Areas at higher latitude as well as along China’s coastline are typically expanding. The expansion of China’s coastline is the result of the large sediment loads carried by the Yellow and Yangtze rivers (carrying the second and fourth largest sediment load^20^ in combination with large-scale land reclamations (over 5000 km^2^ since the year 2000^21^)). The expansion of muddy coasts in North America’s Hudson Bay is likely the result of isostatic rebound leading to sea level fall^22^; the expansion in Europe is related to land reclamations^23^. Many coastlines at lower latitudes experience erosion (North America, Pakistan, India) or alternate between erosion and sedimentation (the Amazon coastline, Indonesia, Bangladesh). Erosion along the Pakistan coastline is primarily driven by a reduction of the sediment load of the Indus (95%^24^). The alternating patterns of erosion and accretion along the northeast coast of South America reflect the natural migration cycles of mud banks^12,25^.

Two out of the five areas with strongest erosion rates are located in Bangladesh (Table 2), where stretches of 13–29 km long muddy coasts have eroded on average between 31.0 and 50.7 m/yr over the study period. The world’s longest muddy coastal stretch suffering severe erosion is located at Pulau Beruit in Malaysia where we observed a 40 km stretch of muddy coast with a mean erosion rate of 19.3 m/year. The largest accretive hot spot is in Indonesia, North Sumatra along the straits of Malacca, at the river mouth of the Rokan River, where a stretch of 17 km of muddy coast has accreted on average 37.6 m/yr. Not only the mudflats seem to have accreted, but also the (mangrove) forest/vegetation has expanded seaward. It is noteworthy that at the north east coast of South America large erosion and accreting hot spots alternate. The Guyana current transports Amazonian sediments (mainly mud sediments) along the north coast of Brazil into French Guyana and Suriname^26^, where mudbanks migrate westward and constantly change shape and orientation due to the combined action of waves and currents^27^.

**Table 2:** World’s largest erosive and accretive muddy coast hot spots

	Areal change rate (m^2^/yr)	Mean change rate (m/yr)	Length of section (km)
Erosive hot spot muddy coasts			
Charfasson, Bangladesh	−889,316	−31.0	29
Pulau Beruit, Malaysia	−772,125	−19.3	40
Johanna Maria, Suriname	−767,178	−55.1	14
Ilha de Maracá, Brazil	−747,625	−37.4	20
Sandwip Island, Bangladesh	−655,014	−50.7	13
Accretive hot spot muddy coasts			
Sinaboi, Indonesia	657,375	37.6	17
Ðông Hung, Tiên Lãng, Vietnam	541,975	41.4	13
Viên An, Ngoc Hien District, Vietnam	489,038	41.2	12
Huian, Quanzhou, China	406,894	26.4	15
North Coast of Friesland, Netherlands	263,347	13.0	20

From a continental perspective, Africa, Oceania and South America are continents subject to net erosion (−0.14 m/year, −0.44 m/year and −0.76 m/year, respectively) whereas the other three continents accrete on average (table in Fig. 2). The continents in the northern hemisphere all accrete on average while the continents in the southern hemisphere are, on average, eroding.

## Methods

**Fig. 3:**
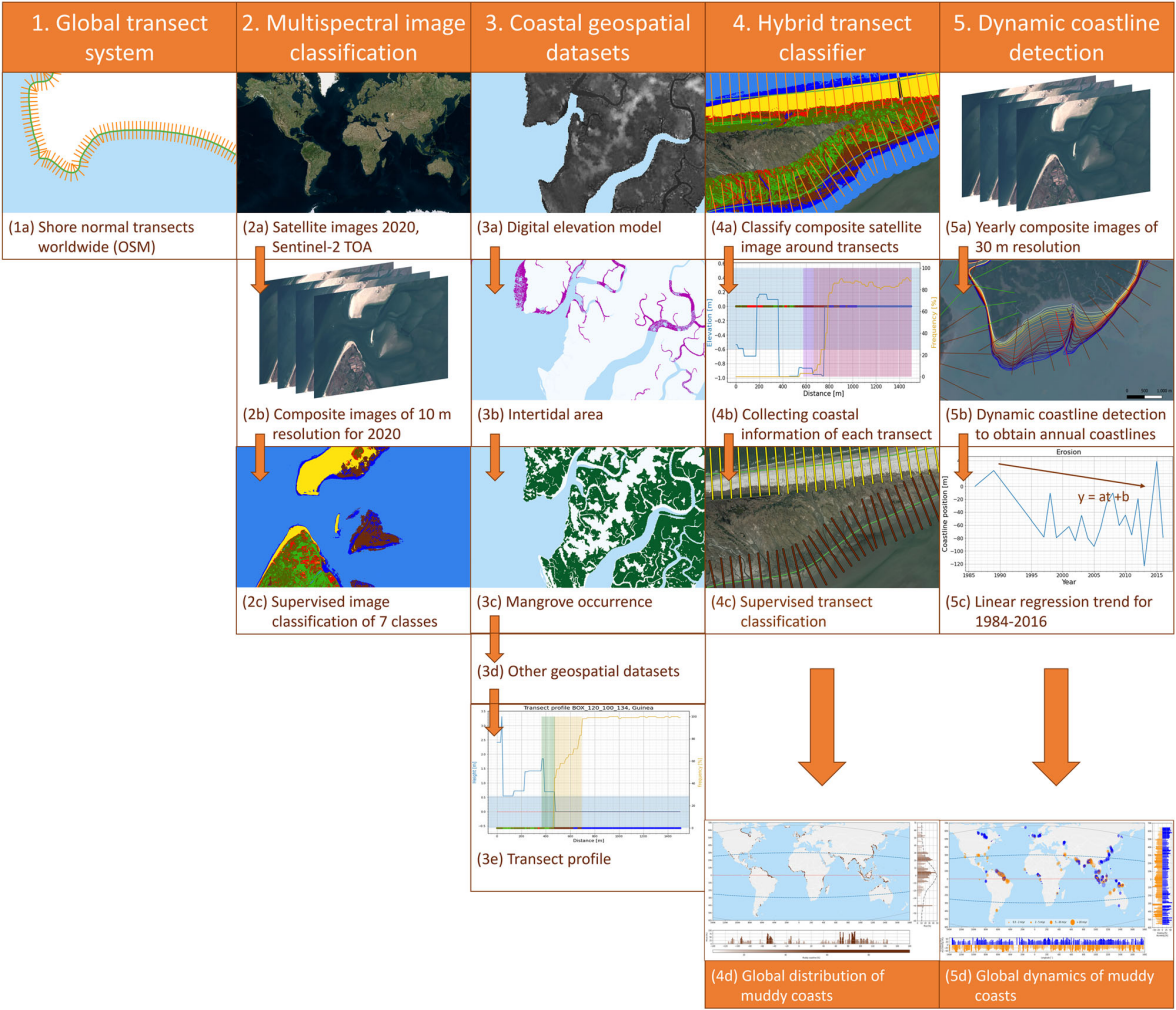
The workflow followed for classifying the global coastline using a global transect system, pixel-based multispectral classification and long-term shoreline changes resulting in global covering data. (1) shows the shore-normal Global Transect System used; (2) shows the steps from Sentinel-2 images to supervised image classification; (3) presents other coastal geospatial datasets used in the hybrid model; (4) presents the steps from the hybrid transect classifier to global distribution of muddy coasts; and (5) shows the steps in computing dynamic coastline changes and upscaling to global scale.

Our hybrid coastal transect classification model (Fig. 3) maps the output of a multispectral pixel-based classifier and global geospatial data on a global coastal transect system^8^. These cross-shore transects are 1500 m long and placed perpendicular to the 2016 global OpenStreetMap (OSM) coastline (https://planet.osm.org/). The spacing of each transect decreases from approximately 500 m close to the equator to 200 m close to the poles (66.5°N and 66.5°S), resulting in a total of 1.8 million transects. In the following sections, we will first explain the pixel-based classification and combination with global datasets into the hybrid classifier, followed by a validation of this methodology.

### Pixel-based multispectral classifier

The pixel-based multispectral image classifier uses freely available Sentinel-2 (S2) multispectral global Top of Atmosphere (TOA) images (https://scihub.copernicus.eu/), to classify coastal-cover and -use of the global coastal area. The S2 Multispectral Instrument (MSI) samples thirteen spectral bands: visible and near infrared (NIR) at a spatial resolution of 10 m, red edge (RE) and short wave infrared (SWIR) at 20 m, and atmospheric bands at 60 m. The water vapour band (B9) and cirrus band (B10) are excluded from the analysis as these bands are most sensitive to moisture in the atmosphere.

First, we created a 1-year composite of the 2020 Sentinel 2 TOA reflectance imagery. This was computed by extracting the 15% percentile from each pixel temporal distribution, following the method described in Donchyts et al.^28^ and Hagenaars et al.^29^. This composite image averages the effect of short timescale changes (e.g., wave and vegetation seasonality, tides, and spurious clouds).

As a next step, subsections of the global pixel distribution are calibrated. For this purpose the global coastal area was divided into ~24,000 square boxes of 20 × 20 km. In total, 75 of these 24,000 boxes, representing different coastal environments on the six continents (excluding Antarctica), were selected for training. In total 3240 training data points (pixels) were manually selected and labelled with a coastal class through visual inspection of the RGB 2020 S2 composite TOA images. The training data consisted of seven clearly distinguishable coastal classes: (1) sandy beaches, (2) mudflats, (3) clear water, (4) turbid (brown) water, (5) green vegetation, (6) dry vegetation, and (7) other (containing clouds, snow, buildings and all other indefinable points). An optimised^30^ random forest^31^ ensemble classifier, implemented in the Google Earth Engine platform^32^ as SmileRandomForest, was trained to the spectral reflectance properties of 3,240 labelled points. The algorithm was implemented using fifteen decision-trees, while other parameters, such as the bagging fraction, were left as default. When trained, the algorithm is able to assign a class label (i.e. sand, mud, water, turbid water, vegetation, dry vegetation, other) to each of the pixels in the multispectral satellite imagery. The global multispectral image classifier is verified and validated using sediment samples in a Dutch estuary, which is discussed in Section Performance.

### Global coastal geospatial data

In parallel to the multispectral classifier, we also analysed the physical geospatial characteristics using global coastal datasets. We selected six freely available coastal geophysical datasets that represents different characteristics (Table 3).

**Table 3:** Overview of global coastal geospatial datasets used in this study

Coastal data type	Coastal geospatial dataset	Spatial resolution	Reference
Elevation	MERIT Digital Elevation Model	90 m	Yamazaki et al.^33^
Temperature	WorldClim V1 Bioclim Dataset	1000 m	Hijmans et al.^34^
Mangroves	Global Mangrove Forests Distribution, 2000	30 m	Giri et al.^35^
Tidal flat width	Global Intertidal Change Dataset	30 m	Murray et al.^11^
Transition zone width	Global Surface Water dataset	30 m	Pekel et al.^37^
Tidal range	Deltares Global Tide and Surge Model	5000 m	Verlaan et al.^38^

The Multi-Error-Removed Improved-Terrain Digital Elevation Model (MERIT DEM)^33^ provides indirect information on the coastal environment. A high and variable bed level represents a rocky coast, while a low (variable) coastal topographic relief is usually related to a river delta and therefore to a supply of sediment to the coasts. Mud is most abundant in areas with high temperature and high rainfall and therefore the humid tropics^18^. However, mud coasts occur globally because of the large variability in mechanisms driving sediment production (relief, climate, and rock type in the river basin) and settling (energetic conditions in the receiving basin). Climatological conditions are extracted from the WorldClim V1 Bioclim dataset^34^. The largest expanses of muddy shorelines are associated either with tropical mangrove systems or temperate salt marshes^17^. The presence of mangrove forests is obtained from the Global Mangrove Forests Distribution^35^. Tidal flats are common in sediment-rich environments where the tidal range is large relative to the typical wave height^36^. Tidal flats may range from sand-dominated to mud-dominated, but are typically a combination with mud-dominated upper flats coarsening in the seaward direction. The occurrence of tidal flats can be estimated from global tidal flat ecosystem maps^11^ and the Global Surface Water (GSW) dataset^37^, which can be interpreted as the intertidal area where dry and wet areas alternate (transition zone). Since the tidal range is a strong indicator for tidal flats occurrence, we include a global tidal dataset (the Deltares Global Tide and Surge Model^38^ (GTSM)).

The coastal geospatial data for elevation, mangroves, tidal flat width and transition zone width is extracted every 10 m along the transects (same spatial resolution as the S2 pixels). A number of scalars are subsequently computed per transect. The elevation data per transect is converted into a maximum bed level and a variance along the profile. The Global Mangrove Forests Distribution dataset is used to determine whether a transect crosses mangrove forests (True/False), independent of the amount of mangroves. The intertidal area dataset is used to compute the tidal flat width along each transect. A water probability profile is drawn along each transect using the GSW dataset, from which the width of the transition zone of land to water is calculated; this transition zone is bounded by 5% to 95% water probability in order to remove the (nearly) converging start and end of the profile. The resolution of the temperature and tidal range data is coarser than the resolution of transects, and therefore the temperature and the tidal range data is interpolated using a nearest neighbour interpolation technique. Per transect the maximum temperature of the warmest month and the minimum temperature of the coldest month are extracted from the temperature dataset. The tidal range is calculated per transect by subtracting the mean lower low water from the mean higher high water. Finally, the longitude and latitude of each transect is defined by the coordinates at the centroid of that transect.

### The hybrid transect classifier: detection of muddy coasts

**Fig. 4:**
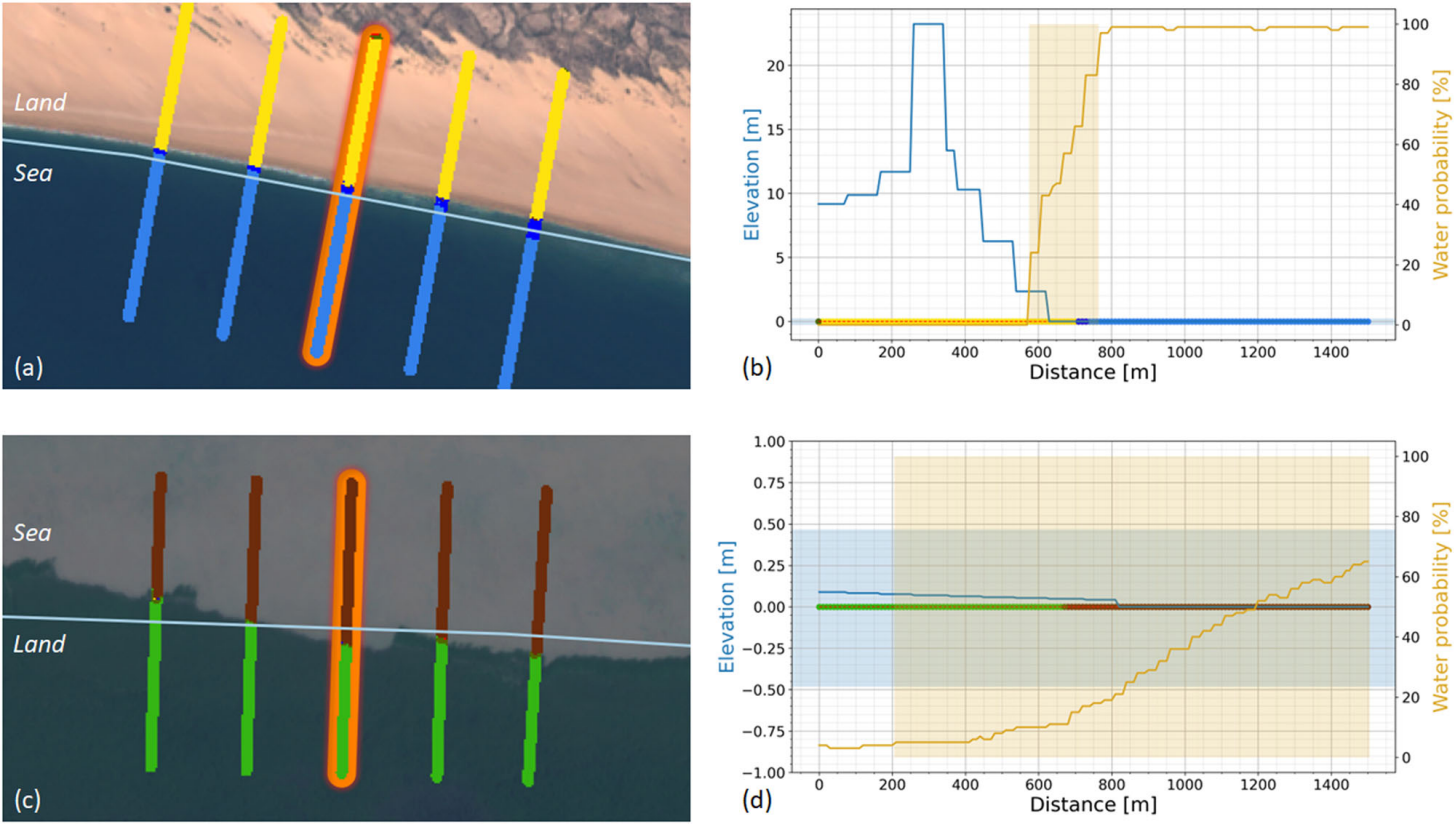
Two examples showing the principle of the hybrid transect classifier. Upper panels **a** and **b**: Madagascar; bottom panels **c** and **d**: Suriname. Left-hand panels **a** and **c**: 2020 S2 composite TOA images with five transects (1500 m in length) each classified by the multispectral image classifier. Colours indicate the multispectral pixel classification. Right-hand panels **b** and **d**: transect profiles of the orange marked transect from the left-hand panel. The horizontal axis is the distance [m] along the transect, where 0 m is the landward end and 1500 m the seaward end. The left *y*-axis represents the elevation [m] above mean sea level to which the elevation profile and the tidal range are related, the right *y*-axis represents the water probability [%]. The multispectral image classification is shown at 0 m elevation in the graphs with coloured dots referring to the coastal class of each pixel along the transect. The tidal range is plotted as a horizontal shaded bar, the transition zone is plotted as a vertical shaded bar.

Both the spectral reflectance properties of land type at pixel level provided by the multispectral satellite images and the physical geospatial characteristics collected with the global coastal geospatial datasets are subsequently integrated into a hybrid transect classifier. The principle of the hybrid transect classifier is illustrated with two examples located in Madagascar and in Suriname, see Fig. 4. The left-hand panels of the figure depict the 2020 S2 composite TOA image, with five transects each classified by the multispectral image classifier. From the classified transects we obtained seven scalars representing the presence of the seven coastal classes along each transect. The right-hand panels of the figure provide indicators from the coastal geospatial datasets in combination with the results of the multispectral classifier. This visualisation allows for an intuitive, contextual inspection of the available information per coastal transect.

In order to train the hybrid coastal transect classification model to identify muddy coasts at global scale, we created a training dataset consisting of 1868 manually labelled transects. These transects are randomly distributed around the globe and capture different coastal environments under different conditions. The training transects are labelled (by one of the authors) by visual inspection of S2 TOA composite RGB images of 2020 along with available high-resolution satellite/aerial imagery (google earth). Five coastal types were defined: (1) sandy coasts (a clear strip of sandy sediment at the coastline is visible on the satellite image); (2) muddy coasts (an exposed tidal flat/mud bank is visible on the satellite image); (3) rocky coasts (large rocky features/cliffs are visible on the satellite image); (4) vegetated coasts (vegetation covers the coastline and no sediment/beach is visible between water and land); and (5) other (all indefinable coasts, including coasts covered with ice/snow, anthropogenic structures that shape the coastline such as harbours and dykes). The transect training dataset consisted of 442 transects associated to each of the four coastal types (i.e. sandy coasts, muddy coast, rocky coast and vegetated coast) whereas 100 transects were categorised as other.

The processed information of the labelled training transects provides a vector, which entries are individual coastal features (i.e. the presence of sand pixels along the transect or the maximum elevation of the topographic profile, etc.). The model consisted (similarly to the pixel land-type classification) as a SmileRandomForest algorithm. The output of the classifier assigned each transect a predicted label with the corresponding coastal type (i.e. sandy coast, muddy coast, rocky coast, vegetated coast, other) that best matched the observed coastal characteristics of the transect. With this classifier all global coastal transects were classified into a certain coastal type.

### Performance

#### Training and accuracy

**Fig. 5:**
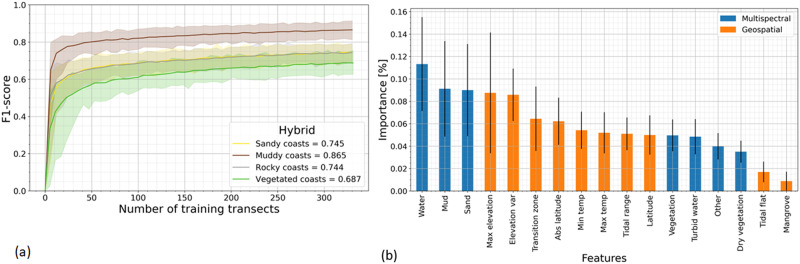
Performance of the hybrid transect classifier and importance of the used features. Left-hand panel **a**: overall accuracy for hybrid transect classifier when validating against the coastal transect types of sandy, muddy, rocky and vegetated coasts. The number of used training transects is on the *x*-axis and the accuracy (f1-score) on the *y*-axis. The solid line represents the mean accuracy, the shaded area represents the 95th percentile of the accuracy. Right-hand panel **b**: importance of features (calculated as the mean decrease in impurity method) used in the hybrid transect classifier in descending order. The bars represent the mean of the importance for each feature originating from the multispectral satellite images (blue) and the coastal geospatial datasets (orange). The black lines represent the standard deviation of the importance of each feature.

Training and testing of both the pixel land-type classifier and the hybrid coastal type transect classifier were done in an analogous manner. First, the manually labelled series were randomly split in a training and testing dataset (75% and 25% respectively), the random forest classifier was trained and its performance evaluated on the test set by means of the overall accuracy and f1-score. This split was performed randomly 100 times in order to describe the variability induced by possible biases in the training-test split. Additionally, subsets of the training dataset of different lengths were used to test the influence of the training data size (see Fig. 5a).

The overall accuracy of the satellite image pixel classification model reached 83.2% for all training points, with the 95% confidence intervals at ±2.5%. The f1-score strongly varies with the coastal class, with water and sand being more accurately resolved than vegetation, mud, or turbid water.

The satellite image pixel classification predictions, along with extracted geospatial datasets at the transect level are used as input for the coastal type transect classifier (i.e. hybrid model). The transect coastal type classifier achieved an accuracy of 76.0% as a mean of all classes. The f1-scores reveal that muddy coast transects are classified most accurately (86.5%; see Fig. 5a). It should be noted that the addition of the geospatial data increased the performance of the transect classifier by 11.2% compared with using information from the multispectral satellite imagery alone (see SM).

The relative importance of features (in the fitted transect classification random forest model) was computed using the mean decrease in impurity method^39^. Figure 5b provides the mean and standard deviation of the importance feature when drawing 100 random splits of the training-testing dataset (75% and 25% respectively). The hybrid transect classifier uses a total of seventeen features of which the three most important originate from the multispectral classifier (water, sand and mud) whereas the following three in order of importance (maximum elevation, elevation variance and transition zone width) are based on the geospatial datasets (Fig. 5b).

The way the classifier is influenced by similar coastal types is visualised in a confusion matrix (see Supplementary Fig. 3). The coastal type that interferes most with muddy coasts are vegetated coasts: although 92 transects (78%) classified as mud coasts were correctly classified, thirteen transects (11%) were in reality vegetated (and seven sandy (6%)). This is probably because the geospatial characteristics of these coasts are very similar, with the exception of (sometimes narrow) mud banks visible on multispectral images. However, 95% of the actual muddy transects were correctly identified by the classifier.

The typical physical geospatial features show differences for the different coastal environments (see Supplementary Fig. 4). Sandy coastlines, for instance, have a larger maximum elevation and narrower transition zone compared to a muddy coastline. These features can therefore be used to optimise the classification. To this end the density distribution of the muddy, sandy, rocky and vegetated coasts are visualised against the geospatial features using the 1,868 labelled training transects (see Supplementary Fig. 4). Muddy coasts generally present a low maximum elevation profile, with a narrow density distribution and a peak at 5 m. The spread of maximum elevation becomes progressively larger for sandy coasts (with a peak at 10 m), vegetated coasts and rocky coasts. Muddy coasts have the widest transition zones, often occupying half the length of the transect. The transition zone width is much narrower for the sandy coasts, rocky coasts and vegetated coasts.

#### Validation

The pixel-based image classification, that fed into the transect classification, has been validated against in-situ sediment observations of sediment grain size distribution in the Dutch Wadden Sea (the Sediment Atlas (https://puc.overheid.nl/rijkswaterstaat/doc/PUC_43681_31/)); see SM for details.

An additional benchmark of the performance of our classifier for muddy coastal transect detection was performed using an independent labelled dataset. To this effect, we utilised the Eurosion coastal typology^40^. This consisted of 4317 coastal transects, which are classified as muddy coastline including tidal flat, salt marsh along coasts in the UK, France and Germany, with a total length of 6936 km. Given the discrepancy in classes, we evaluated the performance of the classifier in terms of true positives (TP/(TP + FN)). When computing the recall over this validation set (*N* = 4376), we find that the classifier has 82% sensitivity, indicating the model’s accurate detection of muddy coasts. See SM for more details.

Also, we conducted a qualitative comparison of the spatial distribution of our muddy areas predicted by our model using a detailed literature survey on the occurrence of muddy coasts along the world’s coastline. The key source of information is the book Muddy Coasts of the World: Processes, Deposits and Function; specifically, Chapter 6: Geographic distribution of muddy coasts^17^. In this chapter a detailed description is presented (of more than 100 pages) on the occurrence of muddy coasts and muddy systems following a stepwise approach along the entire world’s coastline, based on an extensive literature survey.

Following relevant statements in Flemming^17^, we manually drew polygons around the described areas (SM). This resulted in an exclusive digital map of muddy coasts described by Flemming^17^, which was used to qualitatively compare our model-predicted map of the global distribution of muddy coasts (Supplementary Fig. 6). Based on the descriptions by Flemming (2002)^17^, which vary in the level of detail depending on the availability of references, a total of 143 polygons of varying dimensions have been drawn by the authors. Forty-six (46) of the 143 are associated to areas smaller than 50 km or not covered by the global coastal transect system; which can be explained by inland lakes, lagoons, etc. This yields 97 polygons in which significant presence of muddy coasts have been reported by Flemming (2002)^17^; see Supplementary Fig. 7. In 95 of the 97 polygons we also predicted muddy transects. In fact, in 89 of the 97 polygons we found more than 5% of muddy transects. This high agreement (92%) indicates that the model can well predict muddy transects at all muddy coastal regions in the world previously reported in literature, which can be explained by the global spreading of training locations. More details can be found in the SM.

In summary, the data shows to correlate closely to actual coastal muddy areas to the best of our ability across different tests by showing:

1. An accuracy of 86.5% when validating against a global manually curated labelled dataset provided by the authors, using robust metrics that account for test-training size and spatial autocorrelation errors.

2. A recall of 82% when validating against the Eurosion 2004 manually labelled dataset (labelling conducted in 2002 by local geological surveys) for muddy coastlines in NW Europe.

3. A good degree of similarity with the Flemming reported world’s muddy areas, by identifying a large majority of reported muddy area concentrations (a qualitative metric related to the model’s true positive rate).

### Dynamic coastline detection

Shorelines have been detected from Landsat imagery using the coastline detection algorithms of Hagenaars et al.^29^ and the ShorelineMonitor^8^ at a global scale. First, yearly Top of Atmosphere reflectance composites were generated for the period 1984 − 2016, which were subsequently used to estimate an accurate surface water mask using dynamic thresholding method described in Donchyts et al.^28^. Yearly composite images generated by the 15% reflectance percentiles per pixel were analysed to determine global shoreline positions, resulting in the removal of clouds and shadows. This approach is comparable to how Hansen et al.^41^ generates composite images. The use of the composite images significantly decreases the influence of the tidal stage on the detected shoreline positions and averages out seasonal variability in wave and beach characteristics. This dataset on long-term shoreline changes at the global transect system (so for all coastal types) is publicly available at ShorelineMonitor. Combining the classification of all global coastal transects processed in this study, we correlated the classified muddy transects with long-term shoreline changes in the ShorelineMonitor dataset^8^. To avoid unrealistic coastline change rates we applied similar filters as in Luijendijk et al.^8^ to all muddy transects. The muddy transect data is filtered on the number of Satellite Derived Shoreline (SDS) annual data points and the temporal coverage per transect. Transects with possible ice coverage are discarded. In addition, the thresholds for accretion and erosion classes have been increased to 1 m/year to focus on the more dynamic muddy shorelines. In other words, only muddy coasts showing shoreline changes of > 33 m in 33 years are considered in the analyses. Outliers in the SDS are excluded from the linear regression.

The performance of the linear regression method, used to quantify long-term coastline change rates, in capturing trends of chronic muddy coastline change, is validated using three case studies/regions (SM). In addition, the uncertainty bandwidth, which can be considered as a proxy for the representativeness of the linear regression method to express the long-term change rates, is analysed for the muddy transects. This analysis showed that the uncertainties are of the same order of magnitude as for the coastline dynamics of sandy coasts presented in Luijendijk et al.^8^. Even more, the stronger change rates of the muddy transects are more accurate in terms of confidence intervals. We therefore conclude that the adaption of the long-term coastline changes for muddy coasts is justified. Details can be found in the SM.

### Supplementary information

Supplementary Information

## Data Availability

The authors declare that all data supporting the findings of this study is available within the paper and in the Source Data which is archived in a Zenodo digital repository: 10.5281/zenodo.7582197. The data includes the QGIS visualisation, predicted coastal classes at global transects, the manually drawn Flemming (2002) polygons, the transect classification training dataset and the pixel land-type classification training dataset for Sentinel 2.

## Code availability

The code to run the pixel-based multispectral image classifier in this study is archived in a Zenodo digital repository: 10.5281/zenodo.7582197.
