# Supplementary material for: Global distribution and dynamics of muddy coasts
Source: Nat Commun. 2023 Dec 13;14:8259. doi: 10.1038/s41467-023-43819-6 (PMC10716179; doi:10.1038/s41467-023-43819-6)
Supplement: Supplementary file 1 — Supplementary Information [file 41467_2023_43819_MOESM1_ESM.pdf]

Supplementary material
Global distribution and dynamics of muddy coasts

Romy Hulskamp[1,2], Arjen Luijendijk[1,2,*], Bas van Maren[1,2,3], Antonio Moreno-Rodenas[1], Floris Calkoen[1,2], Etiënne Kras[1], Stef Lhermitte[2,4], and Stefan Aarninkhof[2]

[1]Deltares, Boussinesqweg 1, 2629 HV Delft, The Netherlands
[2]Faculty of Civil Engineering and Geosciences, Delft University of Technology, Delft, The Netherlands
[3]State Key Lab of Estuarine and Coastal Research, East China Normal University, Shanghai, China
[4]Department of Earth and Environmental Sciences, KU Leuven, Leuven, Belgium
[*]a.p.luijendijk@tudelft.nl

This supplementary material consists of the following sections:

1. Training transect data selection
2. Validation pixel classification
3. Validation hybrid transect classification
4. Validation of muddy coast distribution using literature survey
5. Validation using Eurosion Typology
6. Limitations in detected muddy transects
7. Uncertainty statistics of shoreline change of muddy coasts
8. Demonstration of annual muddy shorelines
9. Examples of shoreline change time series per class
10. Validation of muddy shoreline change
11. Flemming (2002) quotes and polylines
12. Supplementary References

1. Training transect data selection

Supplementary Figure 1 shows the 1,868 locations of the training transects used to train the hybrid transect classification model.

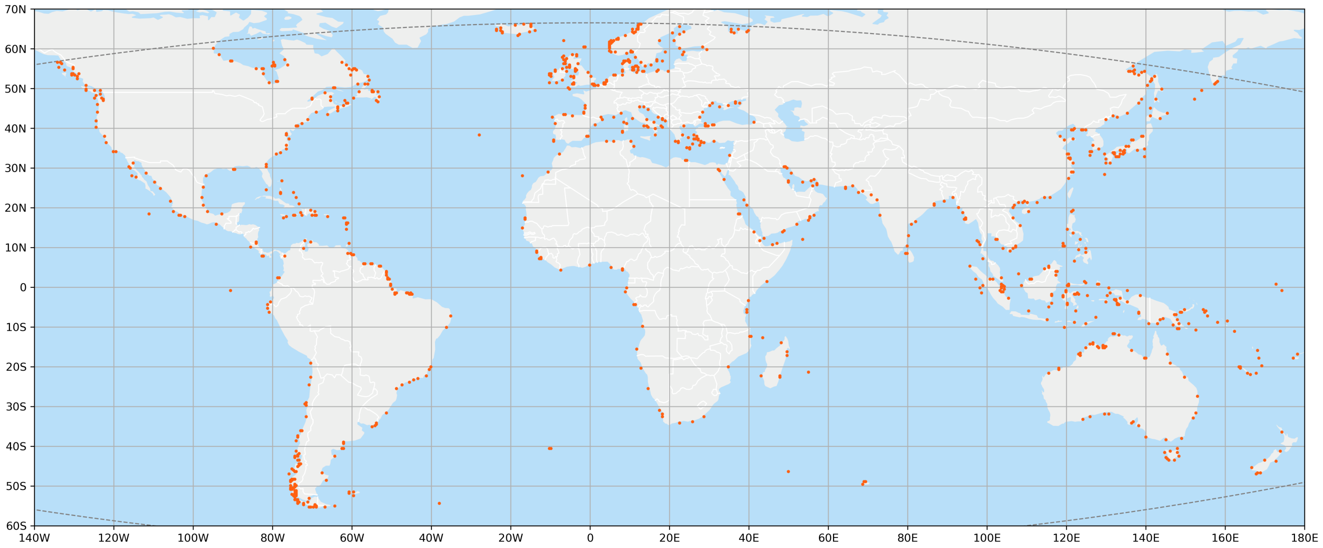

Supplementary Figure 1. Global transect training dataset consisting of 1,868 labelled transects that are represented by the orange dots. The curved grey dashed lines indicate the boundaries of the ice-free coastlines considered in this analysis.

2. Validation of pixel classification

The pixel-based image classification is subsequently validated against in situ sediment observations of sediment grain size distribution in the Dutch Wadden Sea (Rijkswaterstaat, 1998). The exposed coastlines of the barrier islands of the Wadden Sea are sandy, whereas the interior is muddy (in line with observations - see also Alonso *et al.*[1]). The sheltered coastlines, on the mainland coast as well as the barrier island coasts, are sometimes vegetated and not shown here. Our analysis reveals that pixels classified as sand generally have a low mud content, with a peak at approximately 2%. Pixels are classified as muddy for a much wider range in the observed mud content. As a result, 99% of the pixels classified as sandy have a mud content below 25%, while only 64% of the pixels classified as muddy have a mud content exceeding 25%. The reason for low mud content in some pixels classified as muddy is that some sandy beaches are separated from clear seawater by a strip of turbid water which is incorrectly classified as mud.

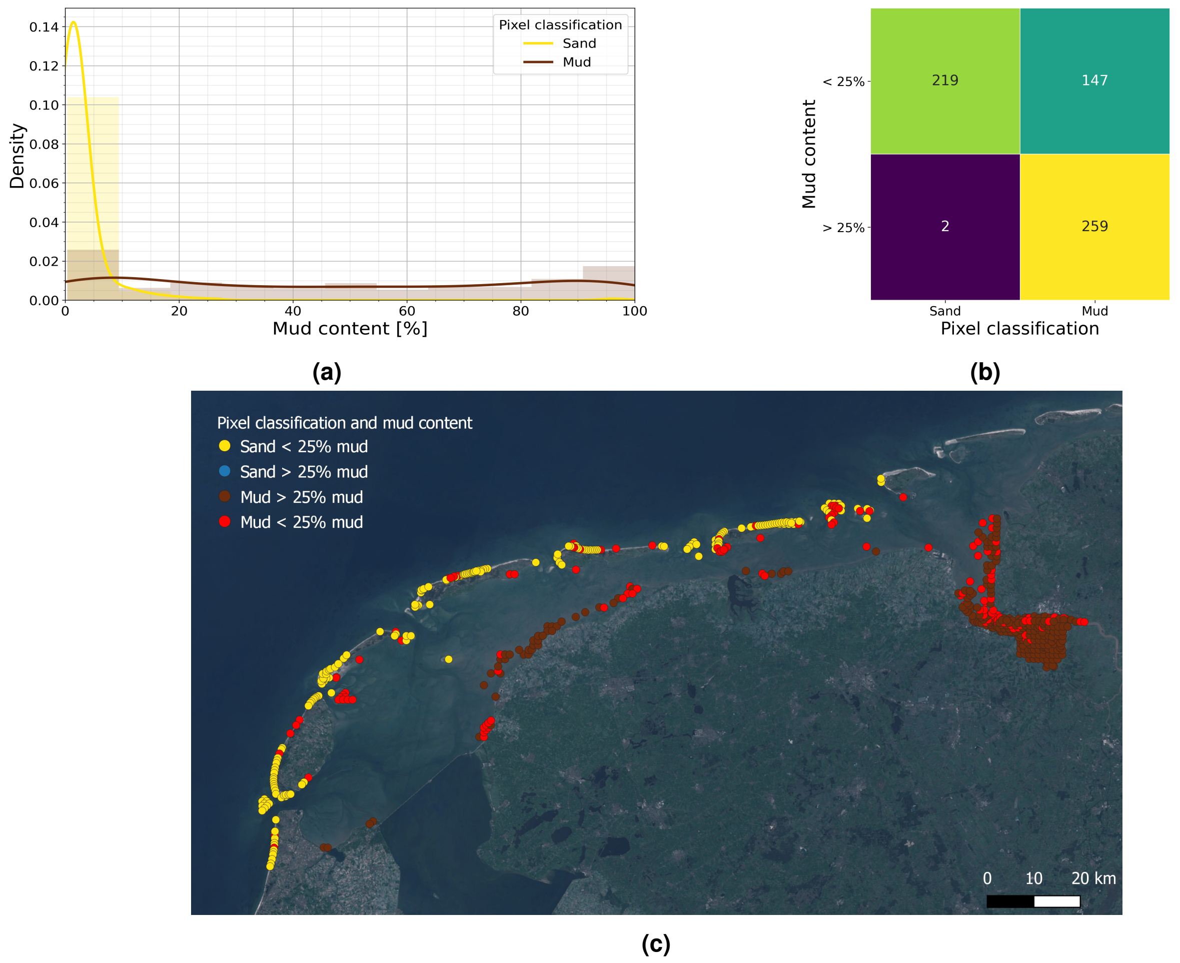

Supplementary Figure 2. (a) Distribution of mud content, among sandy and muddy classified pixels. (b) Confusion matrix of pixel classification and mud content. (c) Map of the pixel classification and mud content of the Dutch Wadden Sea.

3. Validation of hybrid transect classification

For the transect-based performance, 75% of the 1,868 manually labelled training transects are used for training, and 25% for validation. Using the validation transects, we evaluate the accuracy of the multispectral classification method, the geospatial classification method, and the hybrid classification method. The f1-scores reveal that transects are most accurately classified for muddy coasts, and that adding the geospatial data especially improves the classification of rocky and vegetated coasts (compare the top plots in Supplementary Figure 3). The way the classifier is influenced by similar coastal types is visualised in a confusion matrix (see bottom plot in Supplementary Figure 3).

From the values of a confusion matrix the overall model accuracy (OMA) has been calculated using the following equation; OMA=(TP+TN)/(TP+TN+FP+FN); the number of correctly classified validation points (the sum of the diagonal) divided by the total number of validation points.

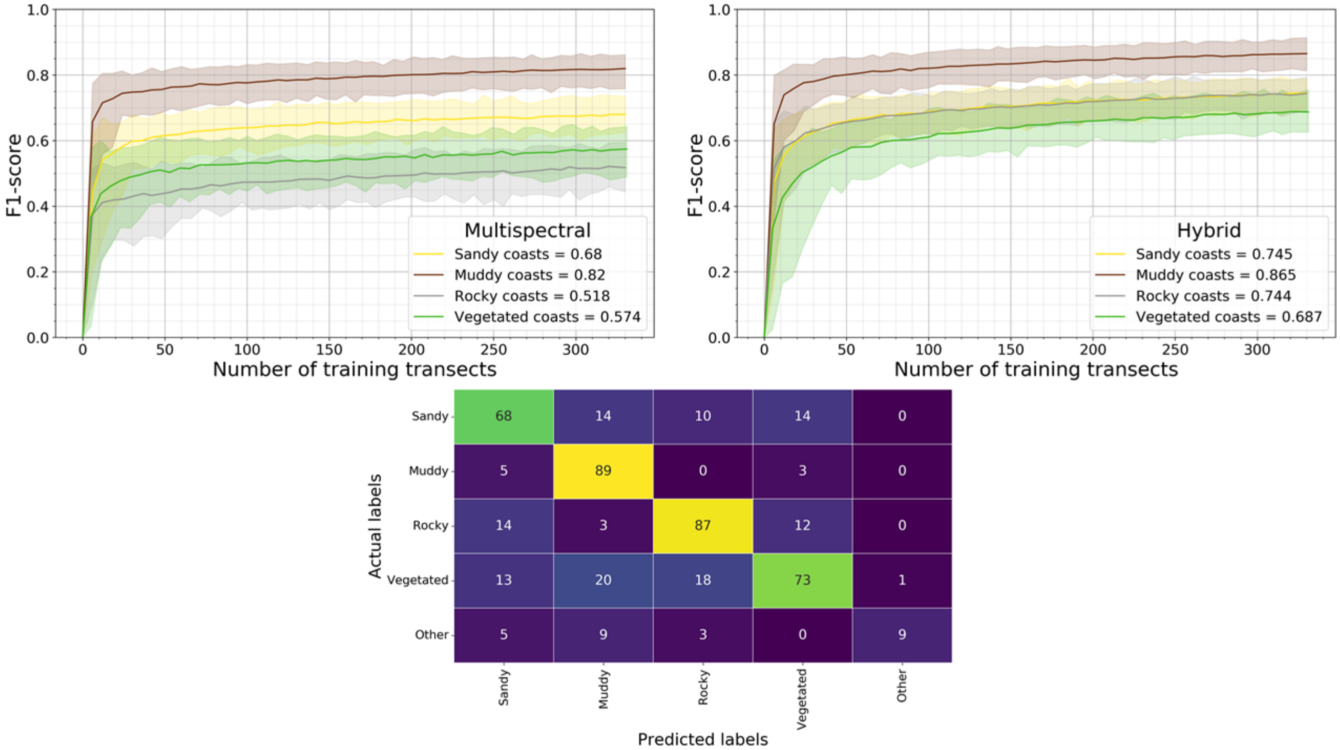

Supplementary Figure 3. Upper left: F1-score for four coastal types (sandy, muddy, rocky, vegetated), when using the multispectral transect classifier. Upper right: F1-score for four coastal types (sandy, muddy, rocky, vegetated), when using the hybrid transect classifier. The number of used training transects is on the x-axis and the overall accuracy (f1-score) on the y-axis. The solid line represents the mean accuracy (f1-score), the shaded area represents the 95th percentile of the accuracy (f1-score). Bottom: Confusion matrix of 25% of the transect training data used for validation. The predicted labels are on the x-axis, the actual labels on the y-axis.

The typical physical geospatial features are characteristic of different coastal environments. These features can therefore be used to optimise the classification. Therefore the density distribution of the muddy, sandy, rocky and vegetated coasts are visualised against the geospatial features using the 1,868 labelled training transects, see Supplementary Figure 4.

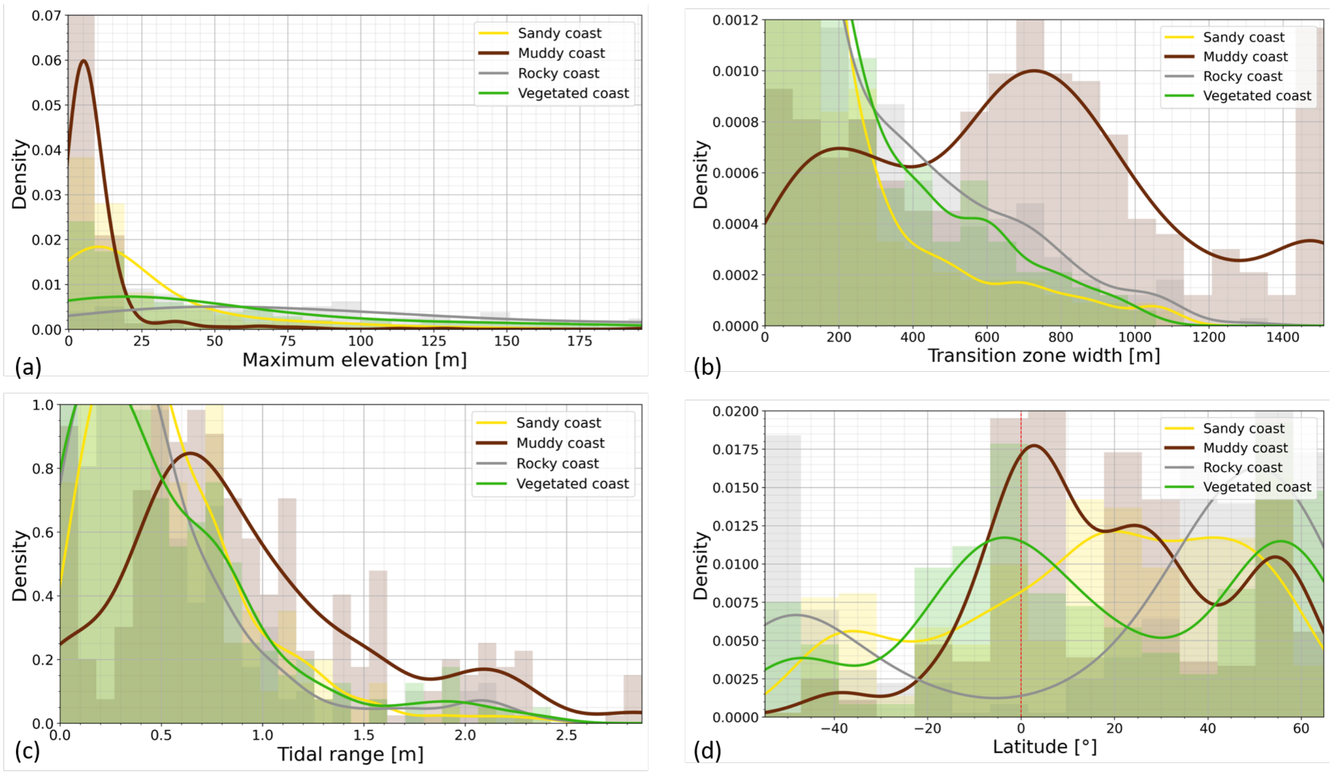

Supplementary Figure 4. Distribution plots for four coastal geospatial variables, based on 1,868 labelled transects. The vertical shaded bars show the (true) densities, the solid lines show the kernel density estimations for the four coastal types: sandy coasts, muddy coasts, rocky coasts and vegetated coasts. (a) Maximum elevation; (b) transition zone width; (c) tidal range; (d) latitude.

Additionally, we tested the influence of the size of the training set in the performance of the predictions by drawing random class-balanced subsamples of different size, along with the dispersion induced by randomly subsampling the training-testing partition (Supplementary Figure 5a). This resulted in an average accuracy of 86.5% for muddy beaches during testing. The spatial dependence of mapped features may result in a biased validation of spatial classifications. This is comprehensively reviewed and discussed in Ploton *et al.* (2020)[2]. To avoid the effect of spatial autocorrelation in the fitting of our model, we first described the spatial structure of muddy labels. This was done by computing the indicator experimental semivariogram for the muddy class and fitting an exponential semivariogram model. This results in a decorrelation distance (range of the fitted exponential model) of 2900 m for the Training set (see Supplementary Figure 5a). In order to verify that this estimate is not heavily influenced by the sparsity of the training set, the same process was also applied in a large subset (N = 100,000 samples) from the world's predicted transects, resulting in a range of 2300 m (see Supplementary Figure 5b).

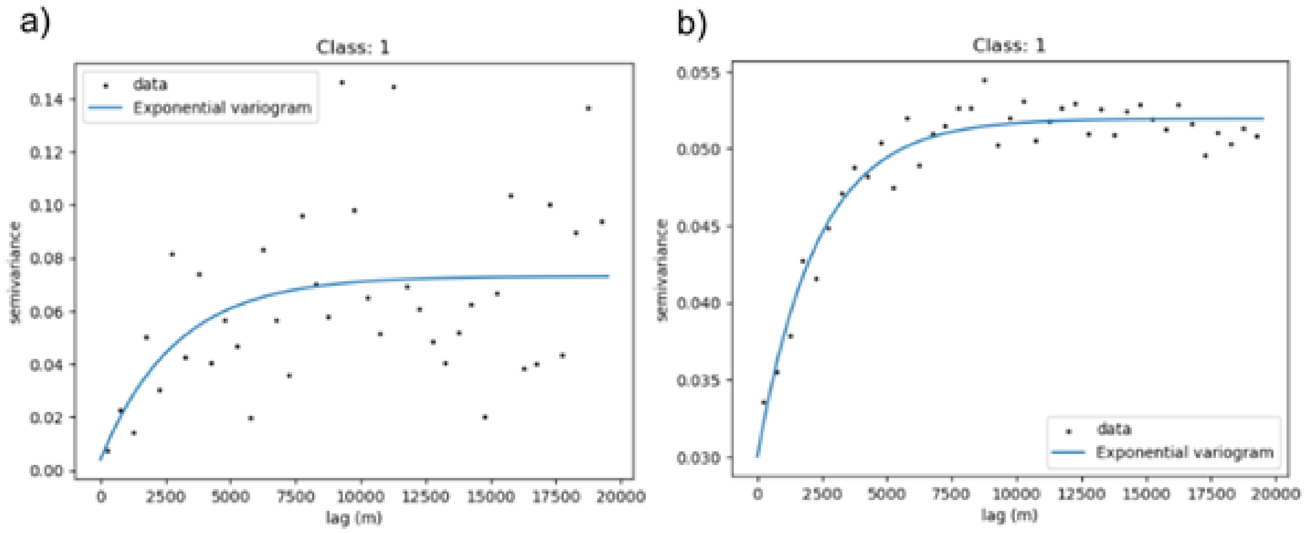

Supplementary Figure 5. Spatial autocorrelation described by an empirical semivariogram and an exponential parametric model for (a) muddy transects from the training dataset (N=1868) and (b) from a subsample of the world's classified transects in our predicted model (N=100,000).

The validation strategy buffered leave-one-out cross-validation (B-LOO CV method, as described by Ploton *et al.* (2020)[2] was applied to avoid the influence of the spatial autocorrelation of muddy areas during the model performance test. To that effect we selected a range of R=3000 m (on the order of magnitude of the spatial decorrelation scale), and iteratively train-tested the model by removing all training points within distance R from the testing transect. This rendered a testing accuracy of 0.88 for muddy zones (and an average 0.73 for all classes), which is consistent with the baseline testing accuracy metrics. Additionally, we tested the sensitivity of the range selection by evaluating [300, 3000, 30000, 300000] m decorrelation ranges, resulting in a test accuracy value for muddy beaches of [0.87, 0.88, 0.86, 0.87] respectively. This insensitivity to the range selected may be explained by spatial consistency of world's muddy beaches features (which the stratified sampling scheme captures well).

4. Qualitative validation of muddy coast distribution using literature survey

To compare the model results an extensive benchmarking with literature has been conducted. This describes the detailed literature survey on the occurrence of muddy coasts along the worlds' coastline. A key source of information is the book 'Muddy Coasts of the World: Processes, Deposits and Function'; specifically, Chapter 6: Geographic distribution of muddy coasts[3]. In this chapter a detailed description is presented (of more than 100 pages) on the occurrence of muddy coasts and muddy systems following a stepwise approach along the entire world's coastline, based on an extensive literature survey.

Below we have copied relevant statements from Flemming (2002)[3] and associated >100 literature references and manually drawn polygons around the described areas. This resulted in an exclusive digital map of muddy coasts described by Flemming (2002)[3], which has been used for validation of our model-predicted map of the global distribution of muddy coasts.

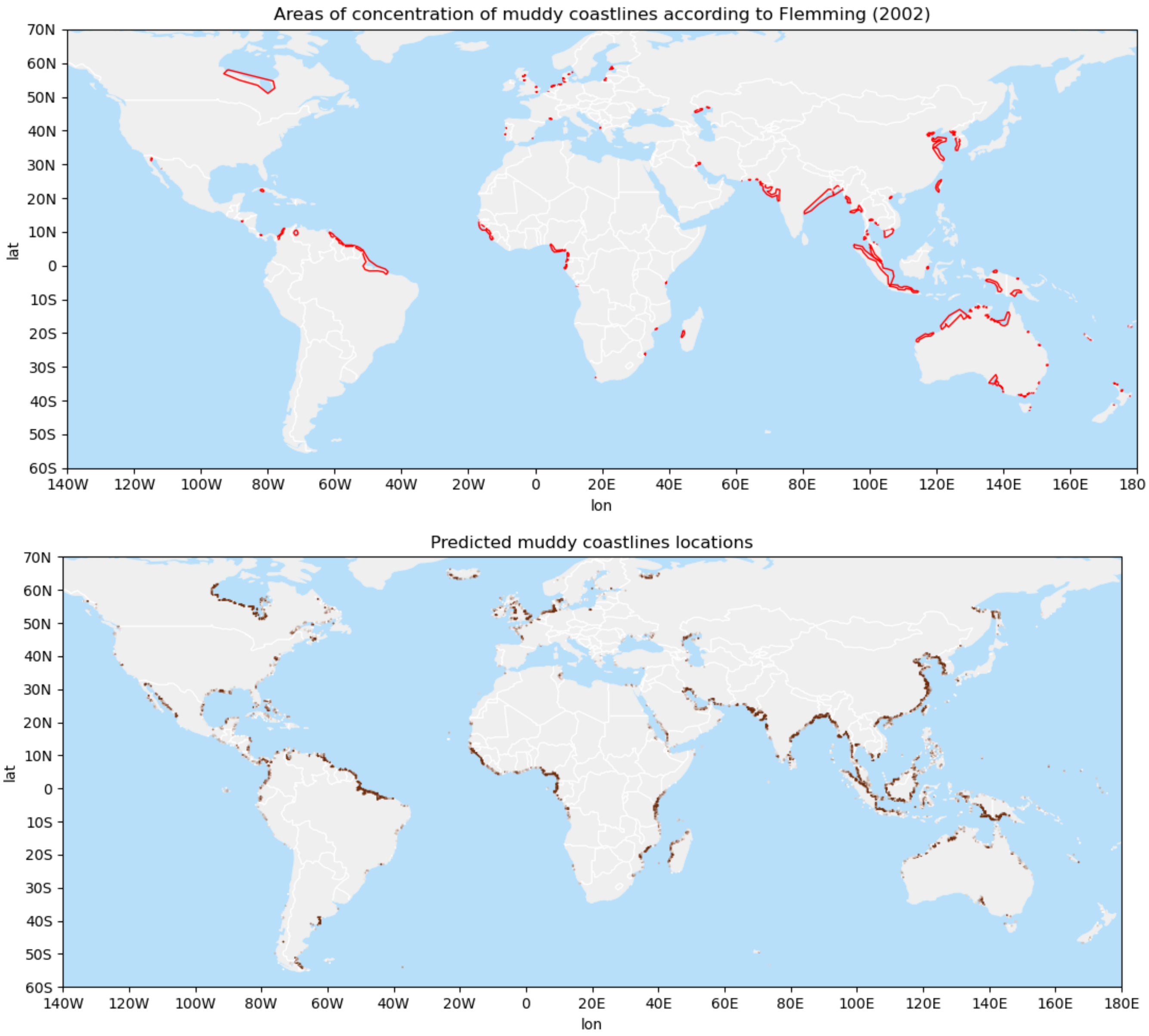

Supplementary Figure 6. Map showing the global distribution of outlined regions where muddy coasts have been reported according to Flemming (2002; top panel) and predicted muddy transects (lower panel).

Based on the descriptions by Flemming (2002)[3], which vary in the level of detail depending on the availability of references, a total of 143 polygons of varying dimensions have been drawn by the authors. Forty-six of the 143 are associated to areas smaller than 50 km or which are not covered by our global coastal transect system; which can be explained by inland lakes,

lagoons, etc.. This yields 97 polygons in which significant presence of muddy coasts have been reported by Flemming (2002)[3]; see top panel in Supplementary Figure 7.

In 95 of the 97 polygons we also predicted muddy transects. In fact, in 89 of the 97 polygons we found more than 5% of muddy transects. This high agreement (score of 92%) indicates that the model can well predict muddy transects at all muddy regions in the world, which can be explained by the global spreading of training locations. Supplementary Figure 7 presents the values for other thresholds for mud only, showing e.g. that in 80 out of 97 (i.e. 82%) regions, up to 10% muddy transects have been predicted. The lower table presents the values for mud + vegetation, showing e.g. that in 96 out of 97 (i.e. 99%) regions, up to 25% muddy transects have been predicted.

Furthermore, our analyses found 39,700 km of muddy coasts within all 89 polygons outlining the areas where Flemming (2002)[3] has reported the occurrence of muddy coasts. In addition, based on the model predictions, we have found a total of 51,700 km of muddy coasts that are located outside the Flemming polygons and hence can be seen as newly mapped muddy coasts.

Threshold for percentage mud (%)	Number of matching Flemming regions (-)	percentage of matching Flemming regions
5	92	95%
**10**	**80**	**82%**
25	66	68%
50	48	49%

Threshold for percentage mud+vegetation (%)	Number of matching Flemming regions (-)	percentage of matching Flemming regions
5	97	100%
10	97	100%
**25**	**96**	**99%**
50	78	80%

Supplementary Figure 7. Relation between percentage mud (upper panel) and mud+vegetation (lower panel) found in Flemming regions and the number of Flemming regions identified.

Highly muddy regions

For each polygon the percentage of muddy transects is calculated. Twenty polygons reveal high percentage of muddy transects (all > 80%), representing a total length of about 12,500 km. When exploring the model results for these highly muddy regions, we regularly observe patches of transects labelled as ‘vegetated’ in a region governed by muddy transects. This can be explained by the fact that at some locations the vegetation directly faces the water when e.g. the beach has been eroded. Following this reasoning, we selected transects labelled as >80% mud and vegetation (assuming mud>60%), and found 38 polygons, adding up to about 42,000 km of highly muddy regions.

5. Validation using Eurosion Typology

To benchmark the performance of our classifier on muddy coasts, we utilized the Eurosion coastal typology[4], which can be found in the EMODnet repository (`https://emodnet.ec.europa.eu/en/geology`). The Eurosion initiative[4] involved the aggregation of geological field data collected by local experts and national geological survey agencies. To ensure a robust comparison, we concentrated on specific geographical areas, namely the UK, German Wadden Sea, and French Atlantic coast (see Supplementary Figure 8). These areas were chosen because they only contain Eurosion field observations – other European regions contain additional observations that have not been validated yet. While the Eurosion coastal typology comprises 26 classes, our classification task focuses specifically on five coastal types. In this validation, we focused on the Eurosion coastal type "muddy coastline, including tidal flat, salt marsh." This decision was made to avoid arbitrary mapping between the 26 Eurosion coastal types and our five coastal types. Our validation process encompassed 4,317 coastal transects, which are classified as “muddy coastline including tidal flat, salt marsh” according to Eurosion (Salman et al., 2004) along coasts in the UK, France and Germany, with a total length of 6,936 km. Given the discrepancy in classes, we evaluated the performance of the classifier in terms of true positives (TP / (TP + FN)). When computing the recall over this validation set (N = 4,376), we find that the classifier has 82% sensitivity, indicating the model’s accurate detection of muddy coasts.

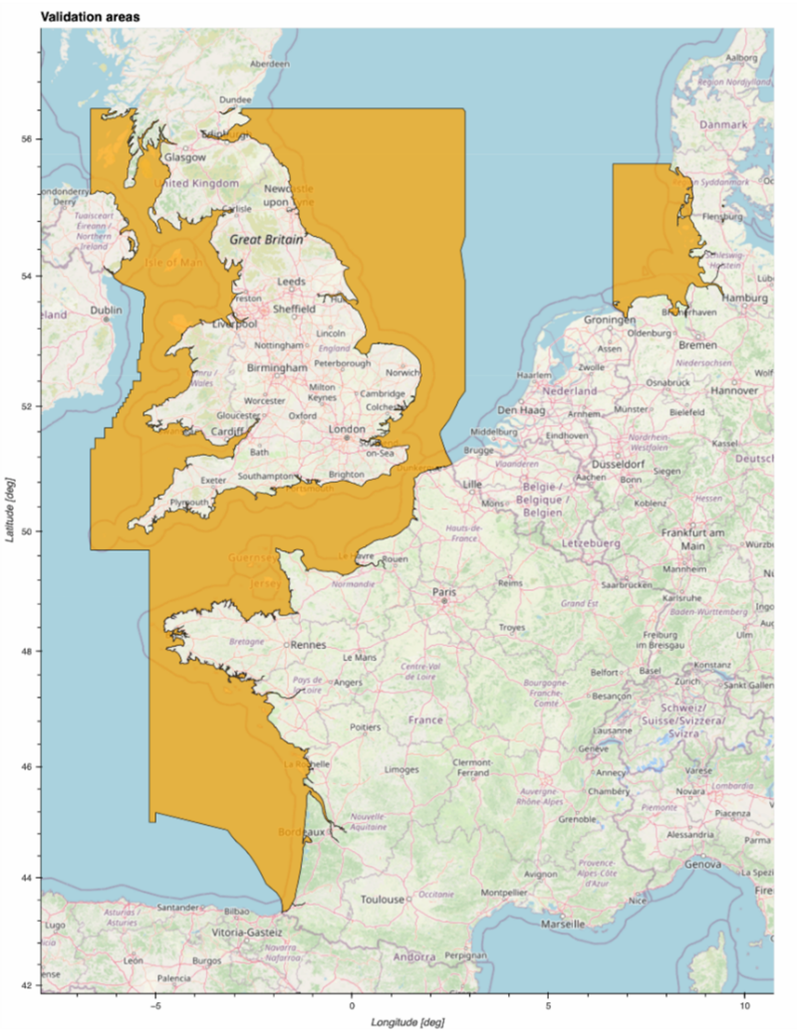

Supplementary Figure 8. Regions used (marked in orange) for validation with Eurosion coastal typology.

6. Limitations in detected muddy transects

In some areas, the model detects muddy beaches where one could argue whether it is indeed muddy. In Iceland we see that muddy transects are predicted inside a small estuary while the sandy transects occur more on the exposed part of the spit (see Supplementary Figure 9). It is not easy to judge whether these are indeed muddy transects in this arctic region.

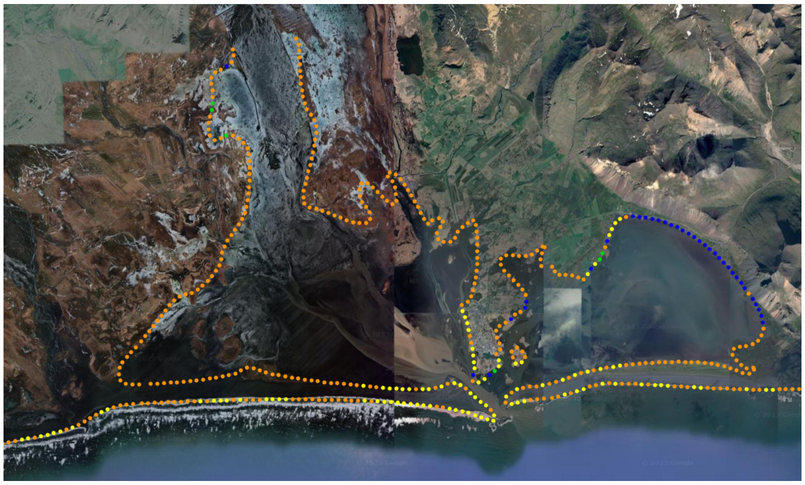

Supplementary Figure 9. Example of the predicted transect labels (yellow = sandy, orange = muddy, green = vegetated, blue = cliff) at the southern coast of Iceland.

At the west coast of Madagascar, sandy transects are predicted at exposed beaches, while at the inner part of the bays mud is predicted (see Supplementary Figure 10). Also here, it is not easy to judge whether these are indeed muddy transects in this tropical region.

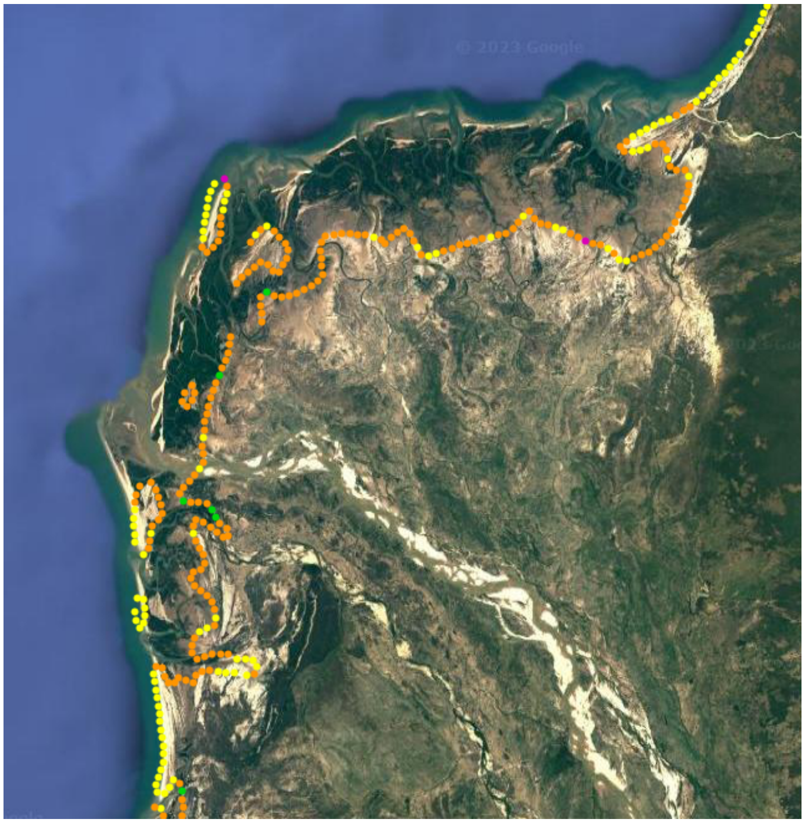

Supplementary Figure 10. Example of the predicted transect labels (yellow = sandy, orange = muddy, green = vegetated, pink = other) at the west-coast of Madagascar.

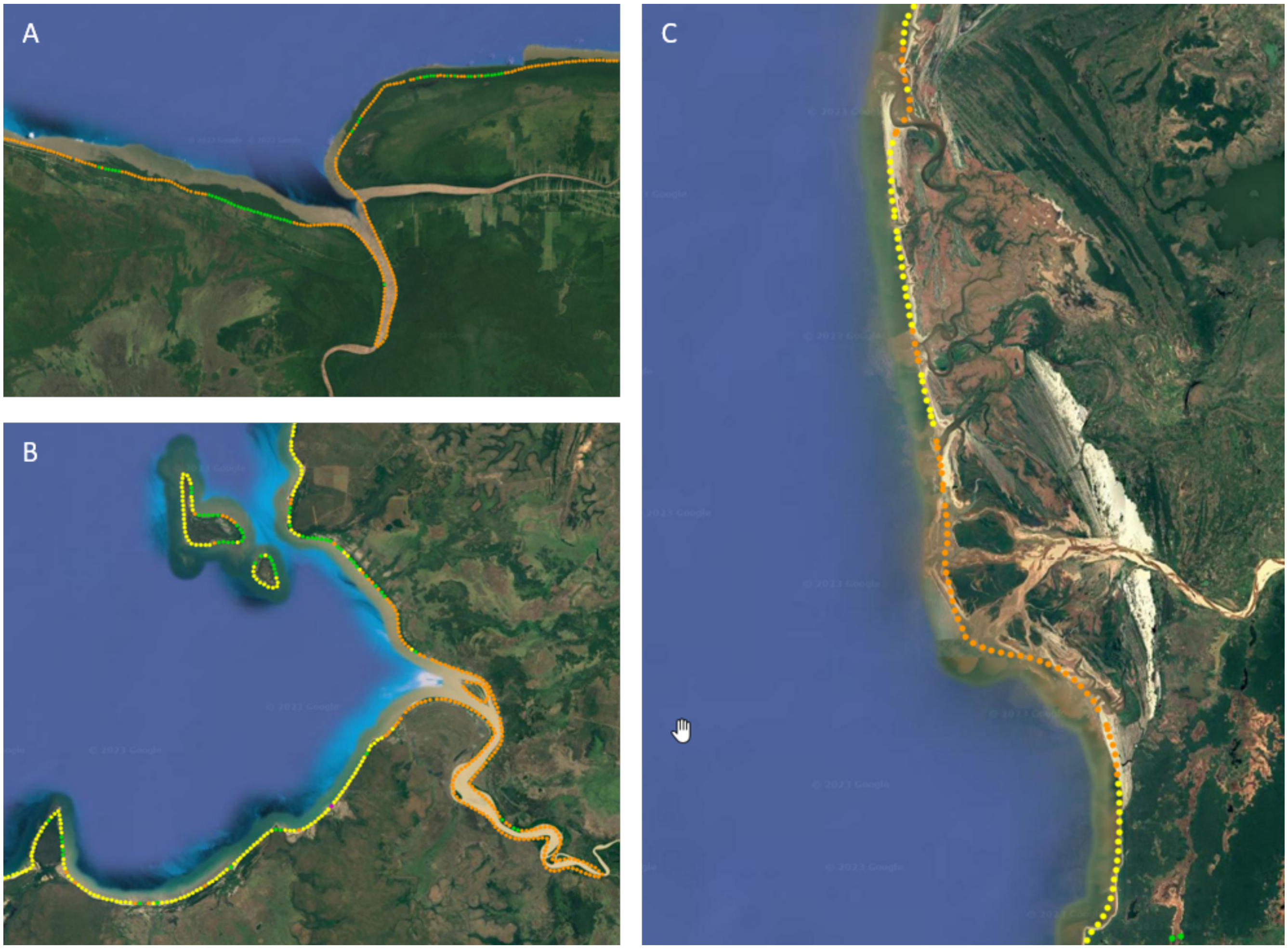

Supplementary Figure 11. Examples of the model predicted transects (yellow = sandy, orange = muddy, green = vegetated). Panel A shows an area at the Amazone coast, where muddy transects alternate with vegetated transects. Panel B shows the clear transition from a muddy river section into a sandy beach environment in the north of Australia, while panel C shows the capability of the model to detect local differences in sediment type at a small river delta and adjacent sandy beaches.

To recap, our model has convincingly identified abundant muddy coasts globally in muddy coastal areas previously reported in literature. On top of that, the model has exposed a significant amount and length of new muddy coasts. Also, on a more local scale we see that the model is able to discriminate between sandy and muddy beach sections (see examples in Supplementary Figure 11).

The global transect dataset is shared through Zenodo (for details see Section: Data availability) and will be presented in an interactive viewer when publishing the manuscript.

7. Uncertainty statistics of shoreline changes of muddy coasts

We assumed that the adaption of the ShorelineMonitor for muddy coasts is justified, because the uncertainties are of the same order of magnitude as for the coastline dynamics of sandy coasts presented in Luijendijk *et al.* (2018)[5]. However, we found that the stronger change rate signals of the muddy transects are more accurate in terms of confidence intervals. Supplementary Figure 12 shows the cumulative distribution function (CDF) plotted against the uncertainty bandwidth for the muddy and sandy coastlines. The change rate uncertainty represents the bootstrapped uncertainty of the linear fit through the time series data of the coastline positions. The uncertainty bandwidth is calculated as the change rate uncertainty divided by the change rate. For example, if a coastline has changed with a rate of 9 m/yr and the uncertainty was 3 m/yr, then the uncertainty bandwidth is 30%. The less uncertainty bandwidth the more accurate the change rate.

The bottom plot in Supplementary Figure 12 reveals that muddy coasts in general have higher change rates (erosion and accretion) than sandy coasts, thus we can conclude that muddy coasts are more dynamic than sandy coasts.

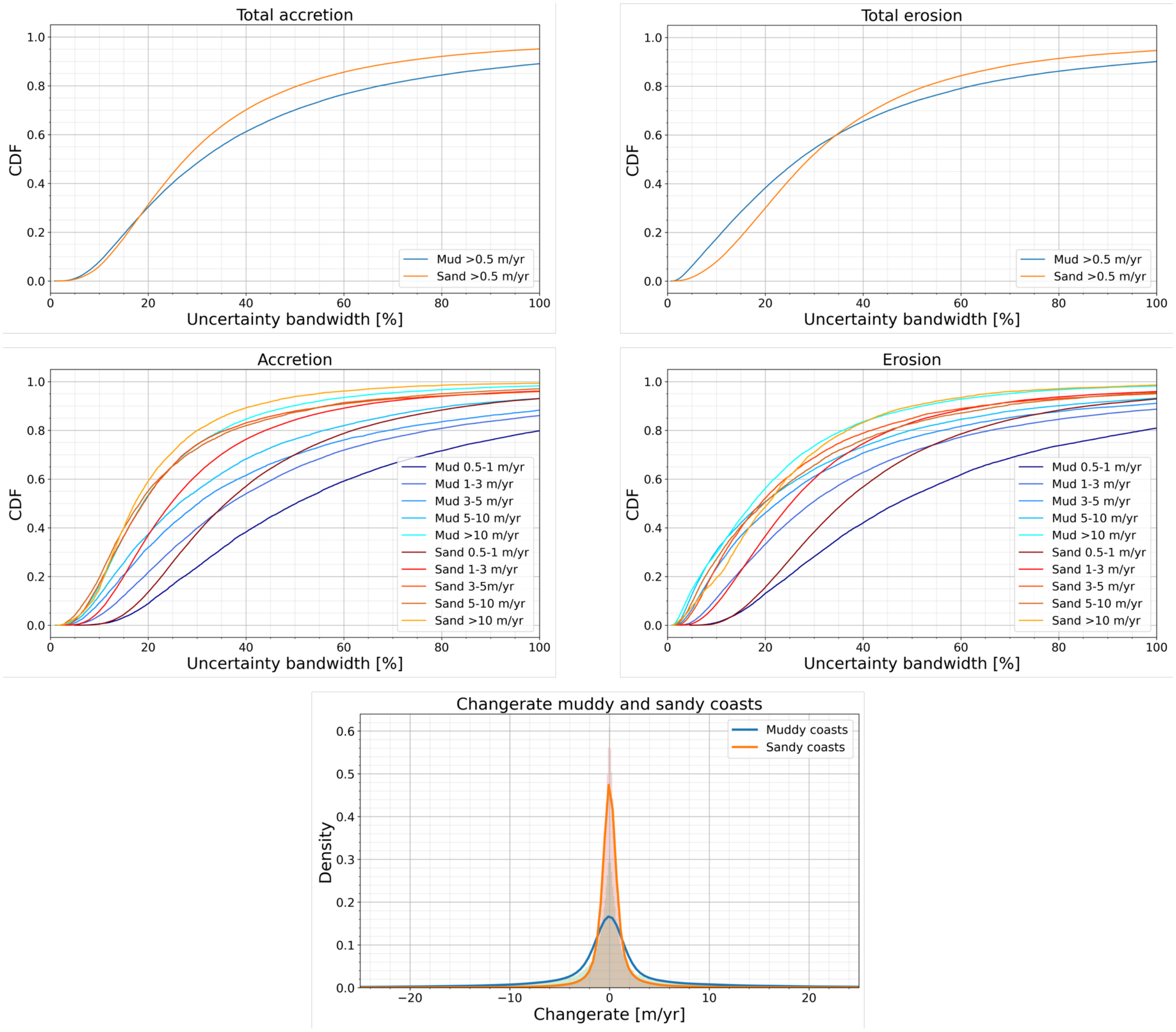

Supplementary Figure 12. Cumulative distribution functions of coastline change rate dynamics for muddy (blue) and sandy (orange) coasts (upper four plots). Change rate density distribution for muddy (blue) and sandy (orange) coasts (bottom).

8. Demonstration of annual muddy shorelines

Here, we demonstrate the dynamics using an expanding and eroding coastline as an example: an eroding muddy coast at the river mouth of the Bidyadhari River in West Bengal, India; an accreting muddy coast at the river mouth of the Pulau River, Papua, Indonesia. The local coastline changes evaluated above reveal realistic trends in erosion and sedimentation.

The river mouth of the Bidyadhari River in West Bengal, India, reveals high erosion rates in the period 1988 and 2016. Erosion rates range between 30-40 m/yr over these three decades. An example of an accreting muddy coast is located at the river mouth of the Pulau River, Papua, Indonesia. This coast is facing the Arafura sea, which is a marginal sea of only 50-80 m depth[6]. The dynamics of this coastline reveals a high rate of accretion in the period 1988 to 2016. Accretion rates range between 20-30 m/yr over these three decades.

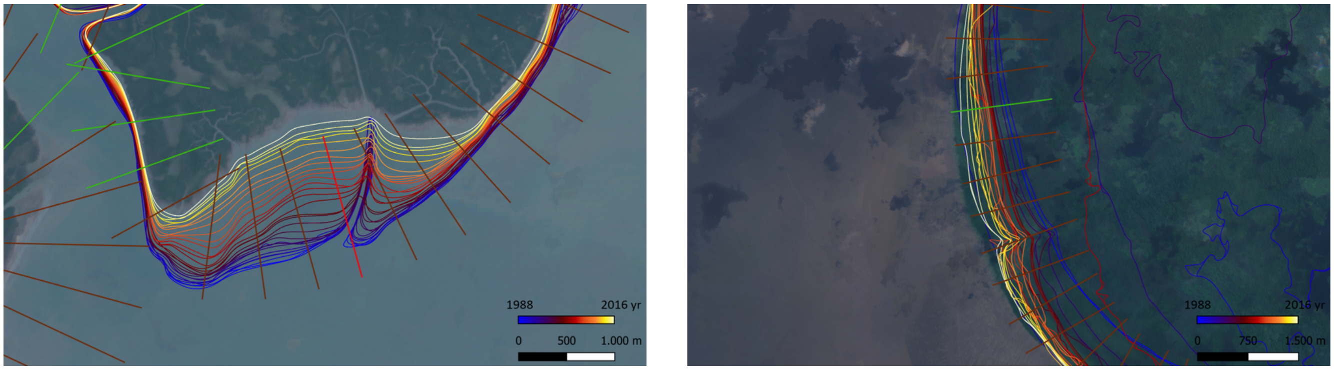

Supplementary Figure 13. Left: Erosive muddy coastline: River mouth of Bidyadhari River, West Bengal, India. Right: Accreting muddy coastline: River mouth of Pulau River, Papua, Indonesia.

9. Examples of shoreline change time series per class

The following Supplementary Figures (14 - 17) present examples of shoreline change time series per dynamic class as presented in Table 1 of the manuscript.

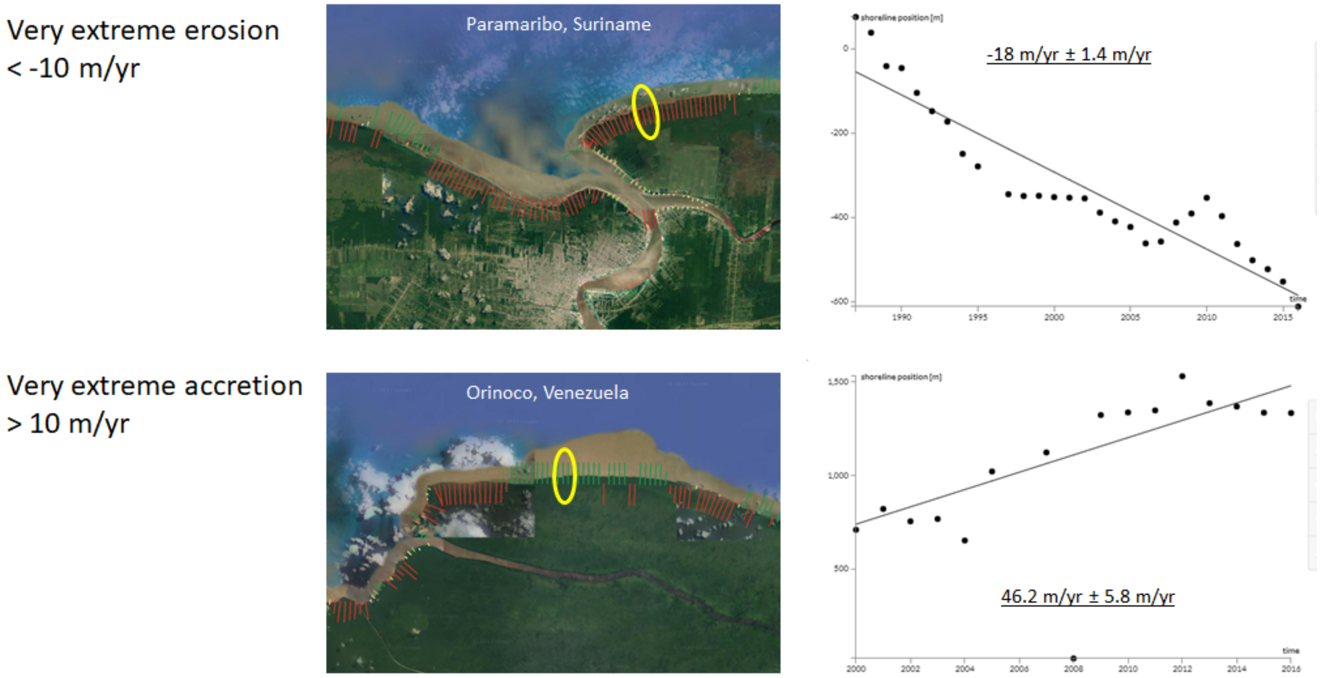

Supplementary Figure 14. Examples of shoreline change time series for the class: *very extreme* dynamics. In the map the green transects represent accretion, red transects erosion. The time series plot presents the shoreline position over time for the transect within the yellow circle.

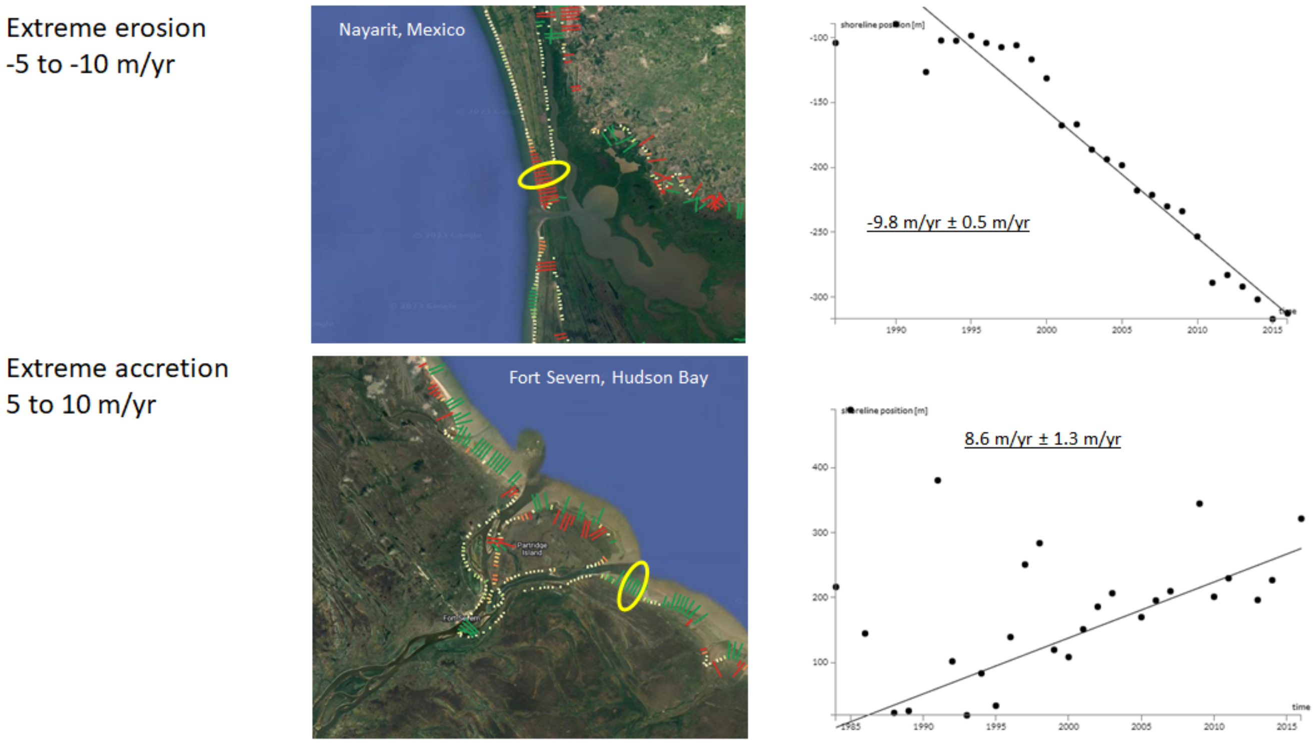

Supplementary Figure 15. Examples of shoreline change time series for the class: *extreme* dynamics. In the map the green transects represent accretion, red transects erosion. The time series plot presents the shoreline position over time for the transect within the yellow circle.

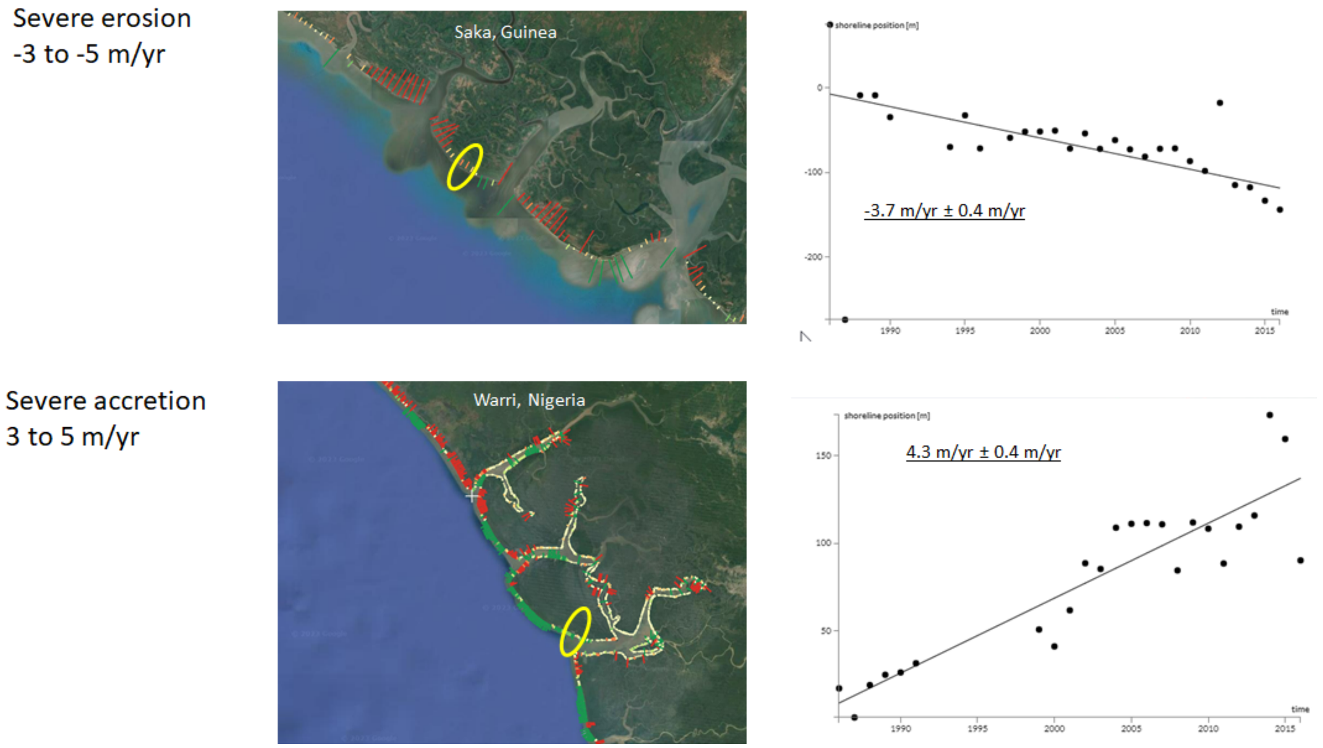

Supplementary Figure 16. Examples of shoreline change time series for the class: *severe* dynamics. In the map the green transects represent accretion, red transects erosion. The time series plot presents the shoreline position over time for the transect within the yellow circle.

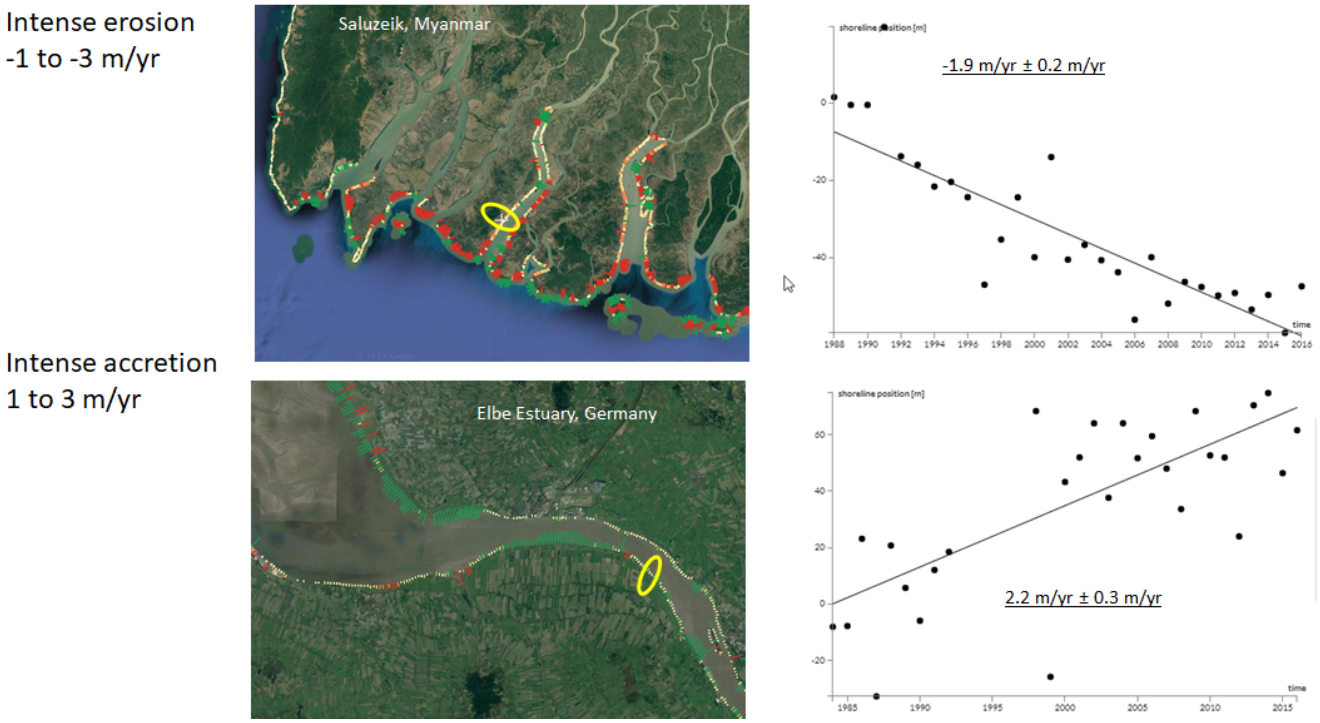

Supplementary Figure 17. Examples of shoreline change time series for the class: *intense* dynamics. In the map the green transects represent accretion, red transects erosion. The time series plot presents the shoreline position over time for the transect within the yellow circle.

10. Validation muddy shoreline change

Literature search resulted in a few papers covering different locations, with varying environmental settings, where either 1) in-situ observations have been used or supported the determination of the reported shoreline changes or 2) some validation has been carried involving ground truthing with field work (e.g. individual shoreline positions). The following 3 sites are elaborated below:

1. Roebuck, Australia

2. Mana, French Guyana

3. Mekong Delta, Vietnam

1. Roebuck, Australia

Mao et al. (2021) presents a dataset for Australia on large-scale decadal shoreline change. A validation of shoreline change at a muddy beach over Roebuck Bay in Western Australia is presented in Fig. 7 in Mao et al. (2021) and shown here in Supplementary Figure 18. Mao et al. (2021) states that both digitization (blue line) and our method (red line) show a similar accretion trend within the AOI (Supplementary Figure 18b). The decadal change rates reported by Mao et al. (2021) for digitization of their shorelines agree reasonably well with our dataset: 0.05 vs. 0.036 km2/yr, contributing to our confidence in the derived long-term shoreline changes in muddy systems.

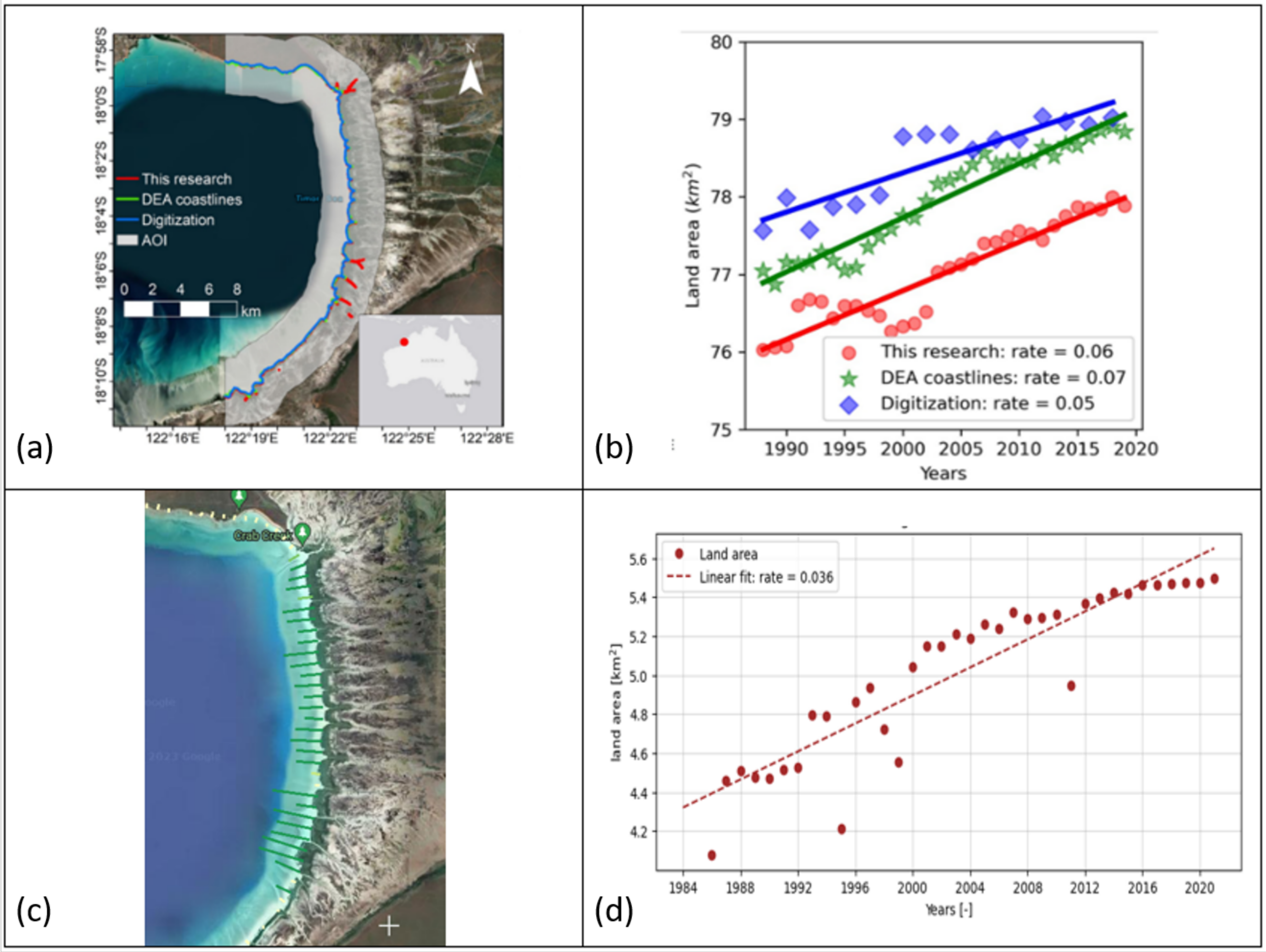

Supplementary Figure 18. (a) Area of interest from Mao et al. (2021) for Roebuck Bay, (b) change in land area (km2) and rates reported by Mao et al. (2021), (c) the area selected to compare the change trends from ShorelineMonitor and (d) represents the corresponding change in land area from the ShorelineMonitor.

2. Mana, French Guyana

Brunier et al. (2019) reports on the exceptional rates and mechanisms of muddy shoreline retreat following mangrove removal in French Guyana. The paper presents the annual shoreline change rates over a stretch of 50km for three distinct periods between 1987 and 2014. They conducted field measurements at a survey site within this coastal stretch to verify their shoreline detection. Supplementary Figure 19 compares the change rates for the three periods spatially for both the reported values in Brunier et al. (2019) and the ShorelineMonitor dataset. Generally, the spatial patterns of erosion and accretion rates agree well for the first and last period. For period 2 some deviations are found around km30 where up to 200 m/yr erosion is reported (hence 1400 m in total) while the ShorelineMonitor shows erosion rates reaching values close to 100 m/yr. Overall, despite some deviations, the long-term spatial and temporal behaviour shows comparable results between the ShorelineMonitor and Brunier et al. (2019).

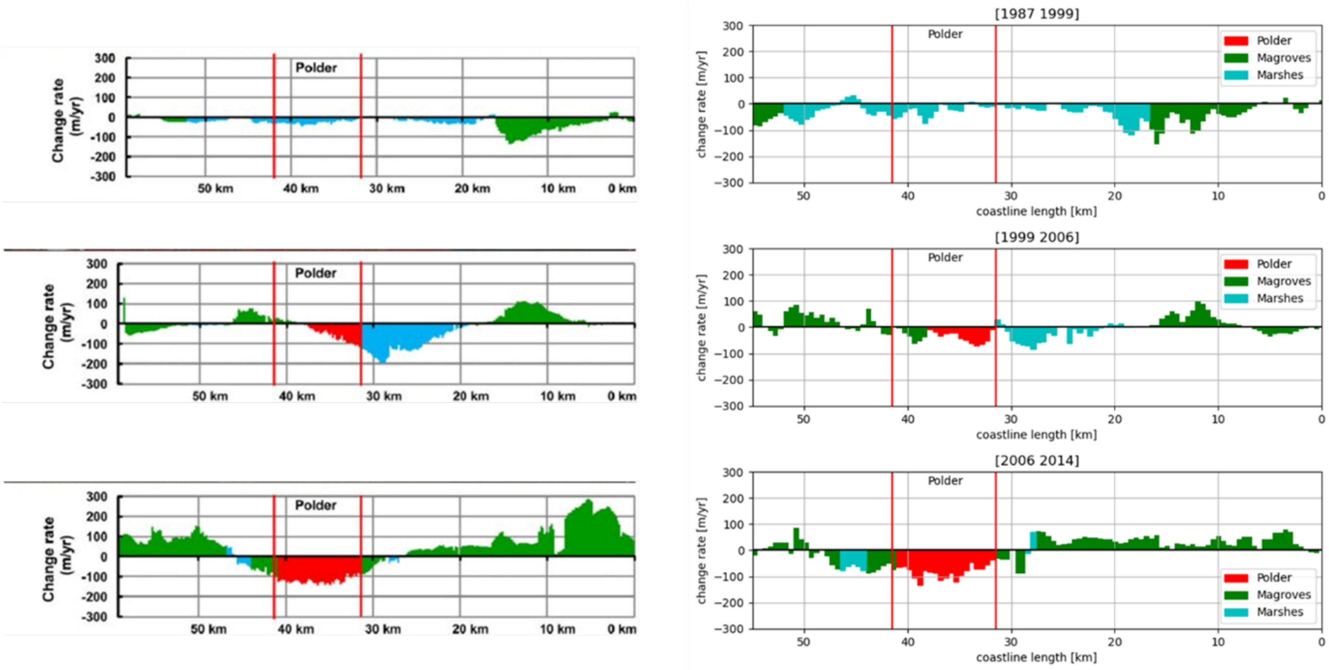

Supplementary Figure 19. Annual change rates reported Brunier et al. (2019) (left column) and ShorelineMonitor dataset (right column) for the period 1987-1999 (upper row), period 1999-2006 (center row), and period 2006-2014 (lower row). Colors of the bars presented in the right column are made consistent with Brunier et al. (2019).

3. Mekong Delta, Vietnam

Anthony et al. (2015) presented a first synthetic analysis of net erosion along the whole LMDCZ (almost 600 km long). Change rates of shoreline (m/year) and coastal area (km2/year) between 2003 and 2012 were analyzed from high-resolution SPOT 5 satellite images. Besset et al. (2016) and Phan et al. (2017) recently renewed this analysis for the period 1973 to 2015 from Landsat images. In the same time, Marchesiello et al. (2019) conducted an investigation for the period 1990–2015, using a combination of Google Earth images, SPOT images and in situ observations. All four studies show local differences but similar patterns of erosion/accretion. In particular, they all found that the Mekong deltaic coast is divided into 4 areas of distinct morphodynamics. The east coast from Soc Trang to Ca Mau showed mostly erosion (Marchesiello et al., 2019). This is illustrated by the two figures from Marchesiello et al. (2019); see upper plots in Supplementary Figure 20. For the same region the long-term change rates from the ShorelineMonitor have been presented showing a similar pattern of erosion and accretion. Where the erosion rates agree well, the accretion rates seem to be overpredicted by the ShorelineMonitor locally (see Supplementary Figure 20).

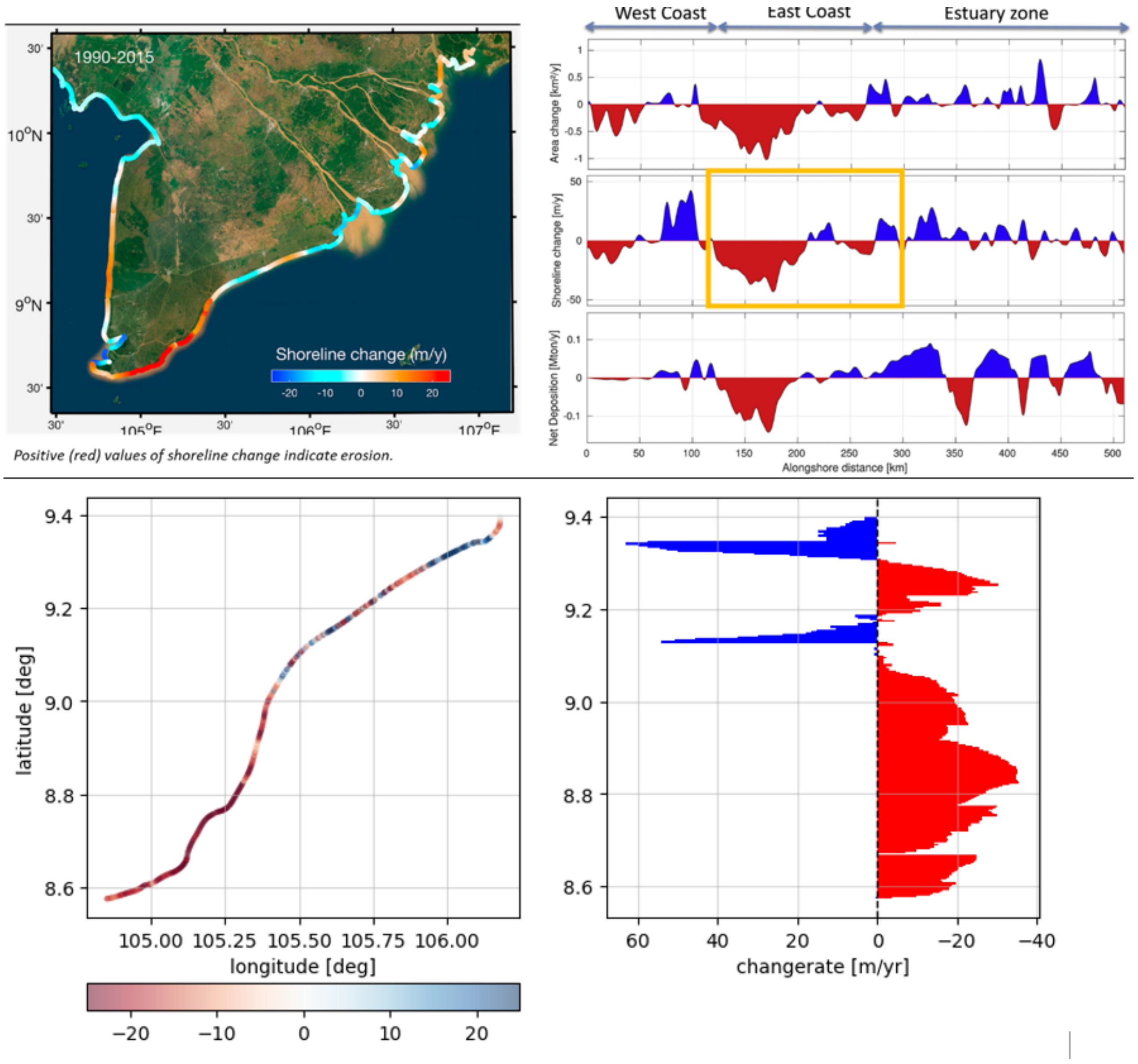

Supplementary Figure 20. Upper left: Spatial map showing shoreline change in Mekong Delta, Vietnam as presented in Figure 16 in Marchesiello et al. (2019). Upper right: Figure 17 of Marchesiello et al. (2019) showing shoreline changes between 1990 – 2015. Lower left: ShorelineMonitor trends on shoreline changes along the East coast of the Mekong Delta. Lower right plot presents the ShorelineMonitor change rates over latitude which can be compared with the sub-figure in the orange box in the upper right plot.

Summary of validation of shoreline change of muddy coasts

The three examples show that the ShorelineMonitor does capture the long-term shoreline trends reasonably well in the three considered muddy systems. Of course, these 3 regions do not fully represent the muddy systems across the world and more validation cases would always be better. Nevertheless, given the good agreements of these validation cases, many visual inspections and the relative smaller uncertainty bandwidths and statistics compared to sandy coasts (as discussed in previous rebuttal), gives us the confidence that presenting the long-term shoreline change trends is justified.

11. Flemming (2002) quotes and polylines

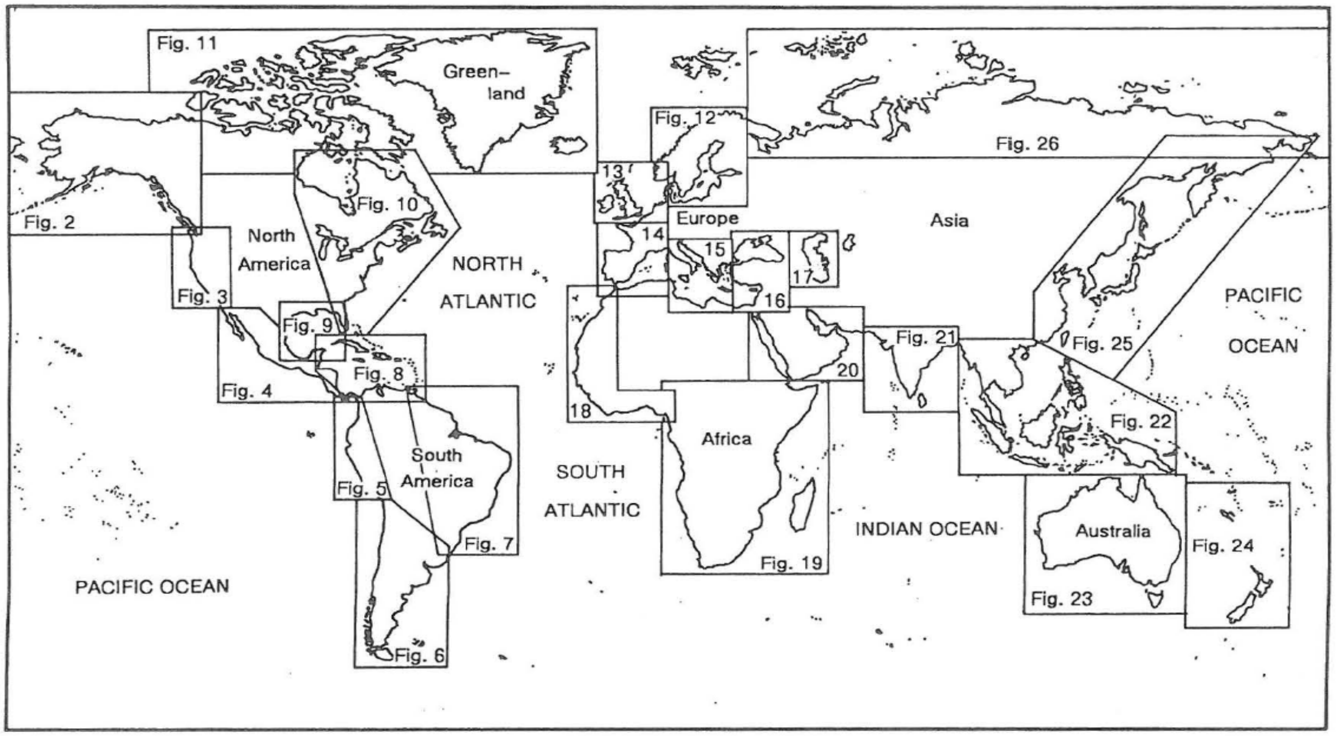

Fig on map	country	Description from Flemming	References	Polygon ID / name
2a	Canada	(British Columbia) is essentially a fjord coast and the only muddy sections of any significance are those formed by the Fraser and Skeena river deltas	Kellerhals and Murray 1969; Luternauer 1980; Swinbanks and Murray 1981; Owens and Harper 1985	Fraser
2b	USA	Muddy shores do not occur along the open Pacific coast, but exist locally within lagoonal estuaries, embayments and river mouths such as Greys Harbour and Willapa Bay in the State of Washington		
4a	Mexico	The largest continuous stretch of muddy coast in this region is found along the north-western shore of the Gulf of California as part of the upper mesotidal Colorado River delta	Thompson 1968, 1975	Colorado River
4b	Mexico	The same applies to the eastern shore of the Gulf, where muddy coastal wetlands are found in Bahia de Adair, Bahia de San Jorge and Bahia Kino, from the delta of the Yaqui River down to Yavaros Lagoon (Estero de Agiabampo) in Sonora Province.	Ayala-Castafiares et al. 1980)	Bahia_Adahir
4c	El Salvador	almost half of the EI Salvador coast is muddy, being partitioned between extensive backbarrier wetlands along its central coastal reach and a mangrove-fringed deltaic environment along the northern shore of the Golfo de Fonseca.		Fonseca
5a	Colombia	With the exception of the long, continuous coastal cliff section north of Cape Corrientes (ca. 300 km), a short cliff section to the north-west of Buenaventura Bay along the central Colombian coast, and a third section near Tumaco in the south, the remaining 900 km of the Colombian coast are fronted by mud flats and mangrove swamps		Colombia - Caribbean
5b	Ecuador	In Ecuador the coastal character changes considerably, muddy shorelines being essentially restricted to three major localities and a number of small estuarinelagoonal sites (Ayon and Jara 1985). The second major mudflat/salt-marsh/mangrove system is developed in the shelter of Cojimies Peninsula. Bordering the Golfo de Guayaquil is the third major and, with a total area exceeding 900 km2, also the largest muddy coastal sector of Ecuador.		Ecuador
7a	Brazil	Between the Maranhense Gulf and the Orinoco delta, situated along the north-east coast of Venezuela, one of the longest continuous stretches of swampy and muddy mangrove-fringed coasts in the world is found	Eisma et al. 1991; Milliman et al. 1982; Meade et al. 1985; Allison et al. 1996	Amazone - Brazil
7b	French Guiana	the coastal plain of French Guiana lies at an average altitude of 4 m and is composed of Holocene marine muds (20% fine silts and 60% clay, the latter predominantly comprising kaolinite) that have been accumulating over the past 8,000 years. Mud bank migration along the coast of French Guiana has been discussed by Froidefond et al. (1988).		Amazone - French Guiana
7c	Suriname	The coast of Surinam is 350 km long and, with a mean tidal range of 1.8 m, is lower mesotidal in character (Psuty 1985). Here the fine-grained coastal plain deposits are 20 km wide in the south-east, widening to a maximum of 140 km in north-west (Zonnefeld 1954). The general topography is dominated by groups of chenier ridges, composed of sand and shell material, separated by wide mud flats (e.g.).	Psuty, 1985; Zonnefeld, 1954; Augustinus 1978	Amazone - Suriname

7d	Guyana	the 434 km long coastline of Guyana consists mainly of mud flats, mangrove forests and chenier ridges	McGill 1958; Schwartz 1985c	Amazone - Guyana
7e	Venezuela	The Atlantic coast of eastern Venezuela north of Guyana is dominated by the Orinoco River delta. It is characterized by extensive muddy, mangrove-fringed interdistributary channels and barrier-lagoons undergoing rapid changes.	(Van Andel 1967; Eisma et al. 1978)	Orinoco
8a	Colombia	From the border with Venezuela to Santa Marta, just south of Cabo San Juan de Guia, the coast is rocky or sandy, being lined by some barrier beaches in its central reaches. The remainder of the coast, up to the border with Panama at Acandi, the coast is extensively lined by mud flats, the shoreward margins being fringed by mangrove swamps.	Schwartz 1985d; Psuty and Mizobe 1982	Colombia - Caribbean
8b	Panama	Caribbean coastline of Panama, Costa Rica, Nicaragua and Belize has received little attention. Indications are that muddy shores are rare and widely spaced	Dengo, 1985	no mud
8c	Panama	The most important of these are Laguna de Chiriqui		Panama - Caribbean
9	Cuba	Thus, along the shores of Cuba, the largest of the Caribbean Islands (cf Bird 1985a), extensive mangrove swamps occupy the entire Peninsula de Zapata along the eastern margin of the Golfo de Bataban6, situated on the south-west coast.		Cuba
9	Bahamas	The Bahama Banks produce huge volumes of carbonate mud.	Geyman et al., 2022	
10	US	It should not go unmentioned that extensive non-marine muddy wetlands occur along parts of the Great Lakes, in particular the north-eastern part of Lake Michigan, the east-central shores of Lake Huron, and the eastern shores of Lake Erie.		Great Lakes
10	Canada	Large upper mesotidal mud flats are also found along the subarctic shores of Hudson Bay and James Bay.	Martini 1991; Ruz et al. 1998	Hudson Bay
11	Iceland	The occurrence of muddy wetlands and tidal flats appears to be insignificant.	Bodere, 1985	
11	Iceland	Arctic salt marsh develops along the muddy intertidal shores of cold regions in arctic Europe, and is known from northern Iceland	EU	
12	Norway	Clayey beach deposits are occasionally found in wave-protected localities where muds are supplied by local streams or where a nearshore mud blanket emerges along a sheltered beach section.		
12	Sweden	Most muddy deposits occur below sea-level and in icedammed lakes.		
12	Finland	Muddy shorelines are restricted to sheltered pocket beaches, where muds winnowed from glacial till have accumulated		
12	Finland	The head of the Gulf of Finland is formed by the muddy marshes and wetlands of the Neva and Luga River deltas, much of the former having been reclaimed in former centuries for the construction of St. Petersburg. Muddy and marshy lowland shores are again found locally along the Estonian Archipelago	Gudelis 1967, 1985	
12	Estland	Muddy and marshy lowland shores are again found locally along the Estonian Archipelago, including the eastern and southern shores of the Islands of Saaremaa and Hiiumaa.		Saaremaa<br>Hiiumaa
12	Estland	The only other muddy wetland and marshy sections along the eastern shore of the Baltic Sea are associated with the sheltered lagoonal shores of the Kursiu Marios and Vistula Bay.	Bartholdy and Pheiffer-Madsen 1985;<br>Bartholdy and Folving, 1986	
13	Denmark	Typical examples in the Kattegat are the Mariager Fjord, where muddy salt marshes line the shore of the estuarine section of the fjord and the Island of Laeso off northern Jylland	Moller, 1985	Island of Laeso<br>Mariagerfjord
13	Germany	the adjacent intertidal flats being today mainly composed of muddy sands with mud contents rarely reaching 50%. This essentially applies to the whole Wadden Sea shoreline between Skallingen in Denmark and Den Helder in The Netherlands.	Biegel and Hoekstra (1995).	Danish Wadden Sea<br>German Wadden Sea N<br>German Wadden Sea W
13	Netherlands		Eisma and Wolf 1980;<br>Veenstra 1980	Dutch Wadden Sea
13	Belgium			Westernscheldt
13	Scotland	A substantial proportion of these intertidal areas comprises mud flats and salt marshes. The three largest estuaries, i.e. the upper macrotidal Solway Firth , the lower macrotidal Tay Estuary and the lower macrotidal Forth Estuary represent well-studied examples.	Perkins and Williams, 1966<br>McManus, 1976<br>Melusky, 1987	Solway Firth<br>Forth<br>Tay
13	Ireland	There are many estuaries with mud flats and salt marshes along the Irish coast, but in general there is little information available on them in the international literature (e.g. Guilcher and King 1961).	Guilcher and King, 1961	
13	England	Some of the better known muddy intertidal environments are The Wash, a lower macrotidal embayment along the east coast of England (Evans 1965, 1975; Evans and Collins 1975), the muddy salt marshes along the coast of North Norfolk (Steers 1960; Pethick 1981), the mudflats along the Thames estuary,	Evans 1965, 1975;<br>Evans and Collins 1975<br>Steers 1960;<br>Pethick 1981	North Folk<br>Thames
14	Portugal	Major estuaries are the Lagoon of Aveiro (e.g. Moreira et al. 1993; Cunha and Moreira 1995), the lagoon at the mouth of the Montego River (Morais 1985), the lower Tagus River or Mar dal Palha which is essentially a river mouth lagoon lined by large mud flats and salt marshes along the south-eastern shore and around the delta of the Tagus River in the north (e.g. Brotas and Catarina 1995)	Moreira et al., 1993<br>Cunha and Moreira, 1995<br>Brotas and Catarina, 1995	Tego estuary<br>Aveiro lagoon
14	Mediterranean	The most notable lagoons and coastal lakes are Mar Menor and Torrevieja just south of the Rio Segura mouth, and Albufera de Valencia south of the City of Valencia.		Mar Menor
14	Mediterranean	Larger muddy swamps are only found in the Rhone delta, especially in the Carmargue (Massoud and Piboubes 1994).	Massoud and Piboubes 1994	Carmargue
15	Albania	The east coast of the Adriatic is mostly steep and rocky, coastal lowlands with a few muddy deltaic deposits only appearing along the coast of Albania, e.g. Karavastas and Narta Lagoons (Shuisky 1985a).	Shuisky 1985a	Narta Lagoon<br>Karavastas

16	Black Sea	Coastal mud deposits are only found on the Cukurova deltaic plain associated with the delta swamps and marshes of the Seyhan and Ceyhan rivers in the south-eastern corner of the country (e.g. Goney 1976).	Goney 1976	
17	Caspian sea	between the Volga and the Ural, and in the large embayment to the east of the Ural River. Due to the aridity of this region, the exposed muddy coastal plain has given rise to the formation of clay dunes		Volga<br>Ural
18	West Africa	As indicated above, the marshy and uniformly muddy shores of the Southern Rivers also occupy the whole coast of Guinea Bissau, the Republic of Guinea, and most of Sierra Leone (Anthony 1997).	Anthony, 1997	Gambia<br>Senegal S<br>Bissau<br>Guinea<br>Sierra Leone
18	Nigeria	the Niger Delta at Mahin where a mud beach is found along the open coast (Ibe et al. 1989). In other places the swales between the ridges form muddy, elongated mangrove swamps which also reach far up into the lower valleys of the Yewa, Ogun, Oshun, and Shasha Rivers. The Niger Delta, which has the same shape as the Volta Delta, except on a very much larger scale, dominates the Nigerian coast by occupying over 60% of the roughly 800 km long coastline. The entire lower delta plain is occupied by muddy intertidal mangrove swamps (Allen 1965a).	Ibe et al., 1989	Nigeria
19	Cameroun	The largest part of coastal Cameroun is lined by muddy mangrove swamps, the only exception being the roughly 50 km section west of the capital Douala which consists of rocky cliffs eroded into the marginal volcanic cone of Mt. Cameroun (Schwartz 1985). The muddy mangrove-lined coast continues past Equatorial Guinea (Rio Muni) into the northern part of Gabon up to the mouth of the Ogooue River	Schwartz, 1985	Cameroun N<br>Cameroun S<br>Equatorial Guinea<br>Gabon N
19	Congo	Large and expanding muddy mangrove swamps are again found on both sides of the Congo River		Congo
19	South Africa	An important coastal wetland along the south-west coast, characterized by extensive mud flats and salt marshes, is Langebaan Lagoon (Day 1959; Flemming 1977).	Day, 1959<br>Flemming, 1977	Langebaan
19	Eastern Africa	The coastal plain conditions observed along the north-east coast of South Africa continue into southern Mozambique up to l6°S, just north of Antonio Enes. This coastal sector is dominated by a number of large rivers such as the Maputo, Incomati, Limpopo, Save, Gorongose, Buzi, Pungue and Zambezi, and landlocked lagoons or lakes in the rear of coastal dune barriers, e.g. Lagoa Pelela (Hill et al. 1975). The rivers carry heavy loads of suspended sediments and are thus associated with extensive muddy mangrove swamps along their lower, estuarine reaches (e.g. Day 1974).	Hill et al. 1975<br>Day, 1974	Zambesi<br>Maputo
19	Madagascar	The central west coast between Cap Saint Andre and the Mangoky delta is smoother, almost the entire coastline being lined by mangroves thriving in the shelter of sandy barrier beaches, especially along the muddy delta lobes of the rivers Manambolo, Morondava, Tsiribihina, and Mangoky.		Madagascar
19	Tanzania	Muddy mangrove swamps occur in the sheltered bays and estuaries of these rivers, but also along the open coast north of the Pagani River. Similarly, most of the islands along the coast (e.g. Mafia, Zanzibar, and Pemba) have flat, swampy valleys along their western shores (Alexander 1985).	Alexander, 1985	Tanzania
20	Iran	The northern Gulf coast of Iran initially remains flat, being characterized by the muddy swamps and marshes of the Zuhr eh Rud delta.		Iran N
20	Kuwait	This coastal type continues into Kuwait and is subsequently displaced by the large deltaic swamps and muddy marshes of the combined Euphrates, Tigris, and Karun Rivers.		Kuwait
21a	Pakistan	Near the Iranian border in the west the mouth region of the Dasht River is formed by a muddy coastal-plain swamp.		Pakistan - Dasht
21b	Pakistan	Further east along the Makran Coastal Region a number of muddy coastal lagoons are encountered, the largest one being Kalmat Khor some 50 km west of Ormara.		Pakistan - Kalmat Khor
21c	Pakistan	Large muddy tidal flats and mangrove swamps, comprising trees of Rhizophora conjugata and Avicennia alba, are found along the shores of Miani Lagoon, situated between Ras Malan and Ras Muari.	Snead and Tasnif 1966	Pakistan - Miani
21d	Pakistan	In the northern, presently inactive delta section tidal channels and mud flats are situated in the lee of sandy barrier bars, whereas open-coast mud flats characterize the active delta further south. Towards the border with India, the delta merges with the Rann of Kutch which forms a vast muddy salt-marsh swamp (e.g. Cori Creek) and which harbours only a few small pockets of mangrove shrubs.	Ahmad 1985	Pakistan - Indus
21e	India	The major part of the Rann of Kutch is situated on the Indian side of the border, the marshy coastline extending into the inner parts of the Gulf of Kutch which forms the northern margin of the Kathiawar Peninsula. The tidal range continues to increase, reaching upper macrotidal amplitudes in the inner Gulf of Khambhat (or Cambay) with 10.7 m at spring tide. As a consequence, the coast is lined by extensive mud flats that are backed by salt marshes and mangrove swamps.	Ahmad 1985	India - Kutch
21f	India	Muddy shores also abound along the east coast of India. In contrast to the west coast major rivers discharge into the Bay of Bengal, several of them, e.g. the Cauvery, the Krishna, the Godavari, the Mahanadi, the Brahmani, and the Ganges, forming large river deltas.	Sambasiva Rao and Vaidyanadhan 1979	India - East
21g	Bangladesh	With the exception of the southern portion of the Chittagong coast, the shores of Bangladesh consist of muddy mangrove swamps or salt marshes.		Bangladesh

22a	Burma	Extensive mangrove swamps occur around river mouths, especially in the coastal sector between the Kaladan River near Sittwe (Akyab) and the Sandoway River mouth 300 km further south.		Burma - Sittwe
22b	Burma	The central coastal reach of Burma is dominated by the Irrawaddy Delta and the Sittang River estuary.		Burma - Irrawaddy; Burma - Sittang
22c	Thailand	They reach their maximum extent between Phuket ad Krabi along the central coastal sector.		Thailand - Phuket
22d	Thailand	Along the east coast of Thailand muddy shores and mangrove swamps are less continuous, being concentrated in the Delta of the Pattani River in the south, on the coastal plain between Songkhla and Nakhon Si Thammarat which is characterized by a number of brakish-water lagoons, along the Ao Ban Don embayments north of Surat Thani, along the coast between Lang Suan and Chumpon, intermittently between Prachuao Khiri Khan and Hua Hin, along the entire north coast of the Gulf of Thailand south of Bangkok, and along the north-east coast between Klaeng and Trat.	Poovachinaranon and Chansang 1982	Thailand - Pattani; Thailand - Ao Ban Don; Thailand - Lang Suan; Thailand - Bangkok; Thailand - Klaeng
22e	Malaysia	Almost the entire west coast is occupied by muddy mangrove swamps, whereas along the east coast the mangrove environment is restricted to sheltered estuaries and the distributaries of the Kelantan River delta.	Ong et al. 1980, 1991; Koopmans 1964; Coleman et al. 1970	Malaysia - West; Malaysia - Kelantan
22f	Malaysia	Mud flats and mangroves also occur on both the west and the east coast of Penang Island which is located along the north-west coast.		Malaysia - Penang West; Malaysia - Penang East
22g	Vietnam	Together with a number of smaller estuaries adjoining the delta to the north-east, this coastal section is extensively fringed by mud flats and mangrove swamps.	Eisma 1985	Vietnam - Mekong
22h	Vietnam	The north-east coast, in turn, is dominated by the actively prograding delta of the Song Hong river (100 m/year).		Vietnam - Song Hong
22i	Indonesia	Very little is known about this coast, other than that vast muddy mangrove swamps dominate the coast.		Indonesia - Sumatra
22j	Indonesia	Again the drainage of the numerous silt-laden rivers is predominantly towards the north. Although the formerly continuous mangrove fringe along the north coast has been largely destroyed, the composition of the coastal plain sediments has (of course) remained muddy.		Indonesia - Java
22k	Indonesia	The only exception is the actively prograding delta of the Mahakam River on the east coast of Kalimantan.		Indonesia - Borneo
22l	Indonesia	These are invariably lined by muddy mangrove swamps as, for example, observed in the Memberamo Delta.	Bird 1985e	Indonesia - Memberamo
22m	Indonesia	Almost the whole south coast of Irian Jaya is occupied by lowland swamps associated with muddy estuaries, e.g. along the lower courses of the Digul, Mapi, and Pulau rivers.		Indonesia - South Papua
22n	Papua New Guinea	Of the larger rivers only the Sepik, located along the north coast, has constructed a large protruding delta.		Papua New Guinea - Sepik
22o	Papua New Guinea	Along the Gulf of Papua on the south coast the main rivers (e.g. the Fly, Wawoi, Turama, Kikori, Purari) open into estuarine channels between which extensive mangrove swamps, backed by nipa palms, are found.	Irion 1983; Thom and Wright 1983; Pickup 1984; Wolanski et al. 1992; Alongi 1991; Alongi et al. 1992; Harris et al. 1993; Baker et al. 1995; Wolanski and Gibbs 1995	Papua New Guinea - South
23a	Australia	Beginning on the east coast of Victoria and proceeding in a clockwise direction, the first muddy coastal environments are associated with a number of microtidal estuaries such as those of the Cann, the Bemm and the Snowy River.	Bird 1985f	Australia - Cann; Australia - Bemm; Australia - Snowy
23b	Australia	Next in line are the Gippsland Lakes located in the rear of Ninety Mile Beach. Since 1889, when an artificial inlet was constructed, these lakes were converted into brakish water lagoons, with former reed swamps having been replaced by salt marsh vegetation.	Bird 1965, 1967b, 1983; Thom 1984	Australia - Gippsland
23c	Australia	The first large estuarine embayment of Victoria is Corner Inlet, situated on the eastern side of Wilsons Promontory. Corner inlet is a shallow marine basin lined by mudflats, seagrass beds, salt marshes and mangrove swamps, Avicennia marina being the only mangrove occurring so far south.	Zhuang and Chappell 1991	Australia - Corner
23d	Australia	Some distance further west in the lee of Cape Patterson, the Tarwin River flows into Venus Bay through Anderson's Inlet. The estuary is line by muddy mangrove belts and salt marshes which are today dominated by the recently introduces Spartina grass.		Australia - Venus
23e	Australia	The west of Venus Bay is Westernport Bay, the second large tidal basin found along the coast of Victoria. The inner parts of this upper mesotidal environment are lined by broad mud flats, salt marshes and mangroves.		Australia - Westernport
23f	Australia	Finally, Port Phillip Bay, at the head of which the city of Melbourne is situated, displays some mud flats and salt marshes near the entrance and in particular on so-called Mud Island which is located near the bay mouth.		Australia - Melbourne
23g	Australia	Just across the border into South Australia muddy salt marsh swamps of limited extend occur along the shores of a string of small coastal lagoons (Lake George, Lake St. Clair, and Lake Eliza) located between two calcarenite ridges between Rivoli Bay and Guichen Bay.		Australia - Rivoli
23h	Australia	In the Coorong barrier system narrow salt marsh belts are associated with carbonate muds in an evaporitic environment.	Von der Borch 1976; Warren 1990	Australia - Coorong
23i	Australia	Similar environments are found in shallow, ephemeral saline lakes or pans along the coast, e.g. Marion Lake at the tip of Yorke Peninsula.	Bolton 1975; Von der Borch et al. 1977; Warren 1982	Australia - Marion
23j	Australia	More extensive intertidal mud flats and supratidal swamps are found along the shores of St. Vincent Gulf and Spencer Gulf which are separated by Yorke Peninsula.		Australia - St. Vincent Gulf; Australia - Spencer Gulf
23k	Australia	Mangroves and mud flats reappear in Exmouth Gulf and continue almost uninterrupted up to the delta of the De Grey River and reappear beyond Eighty Mile Beach to occupy almost the entire lower to upper macrotidal coast of the Kimberly district up to the boundary of the Northern Territory.		Australia - Exmouth; Australia - Eighty Mile Beach

186

23l	Australia	Major rivers with extensive mangrove swamps and mud flats in their estuarine reaches are - from west to east - the Victoria, Daly, Mary, South Alligator, East Alligator, Mann, Goyder, Roper, Limmen Bight, McArthur, Robinson, and Calvert.		Australia - Victoria; Australia - Daly; Australia - Mary; Australia - South Alligator; Australia - East Alligator; Australia - Mann; Australia - Goyder; Australia - Roper; Australia - Limmen Bight; Australia - McArthur; Australia - Robinson; Australia - Calvert;
23m	Australia	Beginning in the north-west, the entire south and south-east coast of the Gulf of Carpentaria is fringed by a wide and uninterrupted belt of muddy mangrove swamps, salt marshes and salt pans reaching northwards up to Cape Keer-Weer along the western shore of Cape York Peninsula.		Australia - Carpentaria
23n	Australia	The large rivers of the region, e.g. the Burdekin at Townsville and the Fitzroy at Rockhampton have built large deltas, the lower estuarine channel systems being lined by muddy salt marshes and mangrove swamps.	Hopley and Murtha 1975; Jennings and Bird 1967	Australia - Burdekin; Australia - Fitzroy
23o	Australia	Typical examples of coastal environments associated with muddy swamps and marshes are the estuary of the Clarence River, Lake Illawarra, Lake Coil, Turron estuary, the lagoons, bays and lakes between Port Macquarie and Sydney (e.g. Wallis Lake, Myall Lake, Nelson Bay, Lake Macquarie, Tuggerah Lake, Broken Bay, Port Jackson, and Botany Bay), the Shoalhaven River delta and Wapengo Lagoon located on the south coast.	Bird 1967b; Roy and Peat 1975; Roy and Peat 1976; Thom 1985l Nichol 1991	Australia - Clarence; Australia - Illawara; Australia - Shoalhaven; Australia - Wapengo
23p	Australia	Along the east coast the Marion Bay barrier shelters well-developed salt marshes in Blackman Bay Lagoon, while similar deposits occur in Moulting Lagoon located in the rear of the Nine Mile Beach barrier.	Davies 1985	Tasmania - Marion; Tasmania - Moulting
24a	New Zealand	Muddy lagoonal swamp or salt marsh deposits associated with headland-bay coasts are found in eastern Northland (e.g. Rangaunu Bay, Doubtless Bay, and Bay of Islands), Auckland (e.g. Firth of Thames), Coromandel (e.g. Mercury Bay), and the Gisborne and Nelson districts (e.g. Poverty Bay).	cf. Woodroffe et al. 1983	New Zealand - Rangaunu; New Zealand - Doubtless; New Zealand - Islands; New Zealand - Auckland; New Zealand - Coromandel; New Zealand - Gisborne; New Zealand - Nelson
24b	New Caledonia	On New Caledonia, for example, muddy mangrove swamps and salt marshes are found in the Bay of St. Vincent, at Mara and in association with the delta formations of the Dumbea, Néra, Poya, Iouanga, and Koumac rivers along the west coast and the Diahot River on the north coast.	Baltzer 1965, 1969; Dugas 1974; Bird and Iltis 1985	New Caledonia - Vincent; New Caledonia - Dumbea; New Caledonia - Néra; New Caledonia - Poya; New Caledonia - Koumac; New Caledonia - Diahot
24c	Fiji	On the Fiji Islands, numerous small muddy mangrove pockets line sheltered embayments and river mouths (e.g. the Sambeto) on Viti Levu, the deltas of the Navua, Singatoka, Nandi, Mba, and Rewa Rivers being particularly good examples.	Bird 1985g	Fiji - Navua; Fiji - Singatoka; Viji - Nandi; Fiji - Rewa
25a	China	From the Yangtze delta to Haizhou Bay, the former mouth region of the Hwanghe River (Yellow River), the coast remains muddy.		China - Yangtze
25b	China	North of Haizhou Bay the muddy shoreline once more becomes embayed due to the irregularity of the coast. This coastal type continues past Qingdao and along the entire Shandong Peninsula up to Laizhou Bay, where the coastal physiography becomes controlled by the modern Hwanghe delta.		China - Haizhou
25c	China	West of the Hwanghe delta, along the shore of Bohai Bay up to the Luan River, the mud flats attain a width of up to 10 km.	Zhao 1980	China - Bohai Bay
25d	China	Coastal marshes and mud flats up to 2 km in width again appear at the head of Liaodong Bay, the source of the mud being the Liao River.		
25e	Taiwan	Muddy shores are found mainly along the west coast of Taiwan, where large amounts of sediment are brought down by local rivers to form wide intertidal sand and mud flats.	Hsu 1965, 1985; Shih 1980	Taiwan
25f	North Korea	Proceeding from the Yalu River delta eastwards into North Korea the coast is initially dominated by wide intertidal mud flats. The tidal range is about 4 m in this region. The mud flats continue up to the mouth of the Taedong Gang.		North Korea
25g	South Korea	Intertidal flats again appear west of Ongjin (North Korea). From here southwards the entire coast is fringed by mud flats of varying width. The west and south coasts of South Korea are extremel irregular, being characterized by numerous embayments formed by intricate headlands of outcropping igneous rocks. Most of these embayments are entirely mud-filled, the tidal flats growing upward and outward from the coast.	Kwon 1974; Wells and Huh 1979; Eisma and Park 1985; Park 1987; Alexander et al. 1990	South Korea
26	Russia	n.a.; coastline is not considered in paper; no ice-free coastline		

187

References related to muddy coasts mentioned in Flemming (2002)

Ahmad, E. 1985. India. pp. 741-748 in: E.C.F. Bird and M.L. Schwartz (editors), The World's Coastline. Van Nostrand Reinhold, New York.

Alexander, C.R., C.A. Nittrouer, D.J. DeMaster, Y.A. Park and S.C. Park. 1990. Macrotidal mudflats of west Korea: a model for interpretation of intertidal deposits. Journal of Sedimentary Petrology 61: 805-824

Alexander, C.S. 1985. Hispaniola. pp. 181-185 in: E.C.F. Bird and M.L. Schwartz (editors), The World's Coastline. Van Nostrand Reinhold, New York.

Allison, M.A., CA. Nittrouer, L.E.C. Faria, Jr., O.M. Silveira and A.C. Mendes. 1996. Sources and sinks of sediment to the Amazon margin: the Amapa coast. GeoMarine Letters 16: 36-40

Alongi, D.M. 1991. The role of intertidal mudbanks in the diagenesis and export of dissolved and particulate materials from the Fly delta, Papua New Guinea. Journal of Experimental Marine Biology and Ecology 149: 81-107.

Alongi, D.M., P. Christoffersen, F. Tirendi and A.I. Robertson. 1992. The influence of freshwater and material export on sedimentary facies and benthic processes within the Fly Delta and adjacent Gulf of Papua (Papua New Guinea). Continental Shelf Research 121: 287-326.

Anthony, E.J. 1997. Evolution of estuarine shoreline systems in Sierra Leone. pp. 39- 61 in: K. Nordstrom and I. Roman (editors), Estuarine Shores: Hydrological, Geomorphological and Ecological Interactions. Wiley Science, London

Augustinus, P.G.E.F. 1978. The Changing Shoreline of Surinam (South America). Natuurwetenschappelijke Studiekring voor Suriname en de Nederlandse Antillen, Utrecht, No. 95, 232 p.

Ayala-Castafiares, A., V.M. Maplica-Cruz and M. Gutierrez-Estrada. 1980. Geologia marina de la region de Yavaros, Sonora, Mexico. Anales Centro de Ciencias del Mary Limnologia. Universidad Nacional Aut6noma de Mexico 2: 81-88.

Baker, E.K., P.T. Harris, J.B. Keene and S.A. Short. 1995. Patterns of sedimentation in the macrotidal Fly River delta, Papua New Guinea. pp. 193-211 in: B.W. Flemming and A. Bartholoma (editors), Tidal Signatures in Modern and Ancient Sediments. Special Publication nternational Association of Sedimentologists 24. Blackwell, London.

Baltzer, F. 1969. Les formations vegetales associees au delta de la Dumbea (NouvelleCaledonie) et leurs indications ecologiques, geomorphologiques et sedimentologiques. Cahiers ORSTOM, Serie Geologie l: 59-84

Bartholdy, J. and S. Folving. 1986. Sediment classification and surface mapping in the Danish Wadden Sea by remote sensing. Netherlands Journal of Sea Research 20: 337-345.

Bartholdy, J. and P. Pfeiffer-Madsen. 1985. Accumulation of fine-grained material in a Danish tidal area. Marine Geology 67: 121-137.

Biegel, E.J. and P. Hoekstra. 1995. Morphological response characteristics of the Zoutkamperslaag, Frisian inlet (The Netherlands) to a sudden reduction in basin area. pp. 85-99 in: B.W. Flemming and A. Bartholoma (editors), Tidal Signatures in Modem and Ancient ediments. Special Publication International Association of Sedimentologists 24. Blackwell, London

Bird, E.C.F. 1965. The evolution of the sandy barrier formations on the East Gippsland coast. Royal Society of Victoria Proceedings 79: 75-88.

Bird, E.C.F. 1967b. Depositional features in estuaries on the south coast of New South Wales. Australian Geographical Studies 5: 113-124.

Bird, E.C.F. 1985e. Indonesia. pp. 879-888 in: E.C.F. Bird and M.L. Schwartz (editors), The World1s Coastline. Van Nostrand Reinhold, New York.

Bird, E.C.F. 1985£. Victoria. pp. 899-911 in: E.C.F. Bird and M.L. Schwartz (editors), The World's Coastline. Van Nostrand Reinhold, New York.

Bird, E.C.F. 1985g. Fiji. pp. 1003-1010 in: E.C.F. Bird and M.L. Schwartz (editors), The World1s Coastline. Van Nostrand Reinhold, New York

Bird, E.C.F. and J. Iltis. 1985. New Caledonia and the Loyalty Islands. pp. 995-1002 in: E.C.F. Bird and M.L. Schwartz (editors), The World1s Coastline. Van Nostrand Reinhold, New York.

Bodere, J.-C. 1985. Iceland. pp. 267-271 in: E.C.F. Bird and M.L. Schwartz (editors), The World's Coastline. Van Nostrand Reinhold, New York

Bolton, B.R. 1975. A geological study of Marion Lake: The site of a Recent stroma to lite occurrence in the Stenhouse Bay region, Yorke Peninsula, South Australia. M.Sc. Thesis, Flinders University, South Australia, 74 p.

Brotas, V. and F. Catarina. 1995. Microphytobenthos primary production of Tagus

Estuary intertidal flats (Portugal). Netherlands Journal of Aquatic Ecology 29: 333-339

Coleman, J.M., S.M. Gagliano and W.G. Smith. 1970. Sedimentation in a Malaysian high tide tropical delta. pp. 185-197 in: J.P. Morgan (editor), Deltaic Sedimentation: Modern and Ancient. SEPM Special Publication 15.

Cunha, M.R. and M.H. Moreira. 1995. Macrobenthos of Potamogeton and

Myriophyllum beds in the upper reaches of Canal de Mira (Ria de A veiro,

NW Portugal): community structure and environmental factors. Netherlands

Journal of Aquatic Ecology 29: 377-390.

Davies, J.L. 1985. Tasmania. pp. 975-979 in: E.C.F. Bird and M.L. Schwartz (editors), The World's Coastline. Van Nostrand Reinhold, New York

Day, J.H. 1959. The biology of Langebaan Lagoon: a study of the effect of shelter from wave action. Transactions Royal Society of South Africa 35: 475-547

Day, J.H. 1974. The ecology of Morrumbene estuary, Mozambique. Transactions Royal Society of South Africa 41: 43-97.

Dengo, G. 1985. Caribbean Central America. pp. 117-124 in: E.C.F. Bird and M.L. Schwartz (editors), The World's Coastline. Van Nostrand Reinhold, New York

Dugas, F. 1974. La sedimentation en Baie de Saint-Vincent. Cahiers ORSTOM, Series Geologie 6: 41-62.

Eisma, D. 1985. Vietnam. pp. 805-811 in: E.C.F. Bird and M.L. Schwartz (editors), The World's Coastline. Van Nostrand Reinhold, New York

Eisma, D. and D.W. Park. 1985. North Korea and South Korea. pp. 833-841 in: E.C.F. Bird and M.L. Schwartz (editors), The World1s Coastline. Van Nostrand Reinhold, New York.

Eisma, D. and W.J. Wolf. 1980. The development of the westernmost part of the Wadden Sea in historical time. pp. 95-103 in: W.J. Wolf (editor), Geomorphology of the Wadden Sea. Balkema, Rotterdam

Eisma, D. and A.J. van Bennekom. 1978. The Zaire River estuary and the Zaire outflow in the Atlantic Ocean. Netherlands Journal of Sea Research 12: 255-272.

Eisma, D., P.G.E.F. Augustinus and C. Alexander. 1991. Recent and Subrecent changes in the dispersal of Amazon mud. Netherlands Journal of Sea Research 28: 181-192.

Evans, G. 1975. Intertidal flat deposits of The Wash, western margin of the North Sea. pp. 13-20 in: R.N. Ginsburg (editor), Tidal Deposits. Springer, Berlin

Evans, G. and M.B. Collins. 1975. The transportation and deposition of suspended sediment over the intertidal flats of the Wash. pp. 273-304 in: J. Hails and A. Carr (editors), Nearshore Sediment Dynamics and Sedimentation. Wiley, London

Flemming, B.W. 1977 Langebaan Lagoon: a mixed carbonate-siliciclastic tidal environment in a semi-arid climate. Sedimentary Geology 18: 61-95.

Goney, S. 1973. Biiyilk Menderes deltasi. Istanbul Universitesi Geografya Enstitii.t Dergisi 18/19: 339-354

Gudelis, V. 1967. Morphogenetic types of the Baltic Sea coasts (in Russian). Baltica 3: 123-145.

Gudelis, V. 1985. Baltic USSR. pp. 303-310 in: E.CF. Bird and M.L. Schwartz (editors), The World's Coastline. Van Nostrand Reinhold, New York.

Guilcher, A and CAM. King. 1961. Spits, tombolos and tidal marshes in Connemara and West Kerry. Proceedings Royal Irish Academy 61B: 283-338.

Harris, P.T., E.K. Baker, AR. Cole and S.A. Short. 1993. Preliminary study of sedimentation in the tidally dominated Fly River Delta, Gulf of Papua. Continental Shelf Research 13: 441-472.

Hill, B.J. 1975. The origin of Southern African coastal lakes. Transactions Royal Society of South Africa 41: 225-240.

Hopley, D. and G.G. Murtha. 1975. The Quaternary Deposits of the Townsville Coastal Plain. James Cook University, Department of Geography, Monograph Series 8, 30 p.

Hsu, T.L. 1965. The tidal flat of the Chiayi area. Bulletin Geological Survey of Taiwan 16: 18-54.

Hsu, T.L. 1985. Taiwan. pp. 829-831 in: E.C.F. Bird and M.L. Schwartz (editors), The World's Coastline. Van Nostrand Reinhold, New York.

Ibe, A.C., L.F. Awosika, A.E. Ihenyen and C.E. Ibe. 1989. Erosion management strategies for the Mahin mud beach, Ondo State, Nigeria. pp. 821-835 in: Anonymous (editor), Proceedings of the Sixth Symposium on Coastal and Ocean Management (Coastal Zone '87), ASCE, New York.

Irion, G. 1983. Clay mineralogy of the suspended load of the Amazon and of rivers in the Papua-New Guinea mainland. Mitteilungen Geologisch-Paliiontologische Institut. Universitiit Hamburg, SCOP/UNEP Sonderband 55: 483-504

Jennings, J.N. and E.C.F. Bird. 1967. Regional geomorphological characteristics of some Australian estuaries. pp. 121-128 in: G.H. Lauff (editor), Estuaries. American Association for the Advancement of Science, Publication No. 83. Washington, D.C.

Kellerhals, P. and J.W. Murray. 1969. Tidal flats at Boundary Bay, Fraser River delta, British Columbia. Canadian Society of Petroleum Geologists Bulletin 17: 67-91

Koopmans, B.N. 1964. Geomorphological and historical data of the lower course of the Perak River (Dindings). Journal Malaysian Branch Royal Asiatic Society 37: 175- 191

Kwon, H.J. 1974. The intertidal flat of the west coast of Korea and the origin of its sediments. Geography 10: 1-12 (in Korean with English summary).

Luternauer, J.L. 1980. Genesis of morphologic features on the western delta front of the Fraser River. pp. 381-396 in: S.B. McCann (editor), The Coastline of Canada. Canadian Geological Survey Paper 80-10.

Martini, LP. 1991. Sedimentology of subarctic tidal flats of western James Bay and Hudson Bay, Ontario, Canada. pp. 301-312 in: D.G. Smith, G.E. Reinson, B.A. Zaitlin and R.A. Rahmani (editors), Clastic Tidal Sedimentology. Canadian Society of Petroleum Geologists emoir 16.

Massoud, Z. and R. Piboubes. 1994. L' Atlas du Litoral du France. Jean-Pierre de Monza, Paris, 332 p.

McGill, J.T. 1958. Map of coastal landforms of the world. Geographical Review 48: 402-405.

McManus, J. 1976. Bottom structures of the Tay Estuary and other estuaries. Scottish

Geographical Magazine 82: 192-197

Meade, R.H., T. Dunne, J.E. Richey, M. de Santos and E. Salati. 1985. Storage and remobilization of suspended sediment in the lower Amazon river of Brazil. Science 228: 488-490

McLusky, D.S. (editor). 1987. Symposium on the NaturalEnvironment of the Estuary and Firth of Forth. Proceedings Royal Society of Edinburgh 93B: 1-567.

Milliman, J.D., J. Butenko, J.P. Barbot and J. Hedberg. 1982. Depositional patterns of modern Orinoco/ Amazon muds on the northern Venezuelan shelf. Journal of Marine Research 40: 643-657.

Möller, J.T. 1985. Denmark. pp. 325-333 in: E.C.F. Bird and M.L. Schwartz (editors), The World's Coastline. Van Nostrand Reinhold, New York.

Moreira, M.H., H. Queiroga, M.M. Machado and M.R. Cunha. 1993. Environmental gradients in a southern Europe estuarine system: Ria Aveiro, Portugal. Implications for soft bottom macrofauna colonization. Netherlands Journal of Aquatic Ecology 27: 465-482.

Nichol, S.L. 1991. Zonation and sedimentology of estuarine fades in an incised valley, wave-dominated, microtidal setting, New South Wales, Australia. pp. 41-58 in: D.G. Smith, G.E. Reinson, B.A. Zaitlin and RA. Rahmani (editors), Clastic Tidal Sedimentologtj- Canadian Society of Petroleum Geologists Memoir 16.

Ong, J.E., W.K. Gong and C.H. Wong. 1980. Ecological Survey of the Sungai Merbok Estuarine Mangrove Ecosystem. Universiti Sains Maylasia, Kuala Lumpur, 83 p.

Ong, J.E., W.K. Gong, C.H. Wong, Z.H. Din and B. Kjerfve. 1991. Characterization of a Malaysian mangrove estuary. Estuaries 14: 38-48.

Owens, E.H. and J.R. Harper. 1985. British Columbia. pp. 11-15 in: E.C.F. Bird and M.L. Schwartz (editors), The World's Coastline. Van Nostrand Reinhold, New York

Park, Y.A. 1987. Coastal sedimentation. pp. 389-405 in: D.S. Lee (editor), Geology of Korea. Geological Society of Korea, Kyohak-Sa Publications, Seoul

Perkins, E.J. and B.R.H. Williams. 1966. The biology of the Solway Firth in relation to the movement and accumulation of radioactive materials. 2. The distribution of sediments and benthos. UK Atomic Energy Authority (Chapelcross, Dumfries, UK) PG Report 587, 37 p.

Pethick, J.S. 1981. Long-term accretion rates on tidal salt marshes. Journal of Sedimentary Petrology 51: 571-577.

Pickup, G. 1984. Landforms, hydrology and sedimentation in the Fly and lower

Purari, Papua New Guinea. pp. 1-17 in: AP. Schick (editor), Channel Processes - Water, Sediment Catchment Controls. Catena Supplement

Poovachinaranon, S. and H. Chansang. 1982. Structure of Ao Yon mangrove forest (Thailand) and its contribution to the coastal ecosystem. pp. 101-111 in: A.Y. Kostermans and S. Matsumoto (editors), Proceedings of the Biotrop Symposium on Mangrove Forest Ecosystem Productivity in South East Asia. Biotrop Special Publication 17.

Psuty, N.P. and C. Mizobe. 1982. Central and South America, coastal ecology. pp. 191-201 in: M.L. Schwartz (editor), The Encyclopedia of Beaches and Coastal Environments. Hutchinson Ross, Stroudsburg.

Psuty, N.P. 1985. Surinam. pp. 99-101 in: E.C.F. Bird and M.L. Schwartz (editors), The World's Coastline. Van Nostrand Reinhold, New York.

Roy, P.S. and C. Peat. 1975. Bathymetry and bottom sediments of Lake Illawarr. Records of the Geological Society of Neu.1 South Wales 17: 65-79.

Roy, P.S. and C. Peat. 1976. Bathymetry and bottom sediments of Tuross estuary and Coila Lake. Records of the Geological Society of New South Wales 18: 103-104.

Ruz, M.-H., M. Allard, Y. Michaud and A. Bequette. 1998. Sedimentology and evolution of subarctic tidal flats along a rapidly emerging coast, eastern Hudson Bay, Canada. Journal of Coastal Research 14: 1242-1254

Sambasiva Rao, M. and R. Vaidyanadhan. 1979. Morphology and evolution of Godavari Delta, India. Zeitschrift ftir Geomorphologie 23: 243-255

Schwartz, M.L. 1985c. Guyana. pp. 103-104 in: E.C.F. Bird and M.L. Schwartz (editors), The World1s Coastline. Van Nostrand Reinhold, New York.

Schwartz, M.L. 1985d. Caribbean Columbia. pp. 115-116 in: E.C.F. Bird and M.L.

Schwartz (editors), The World 1s Coastline. Van Nostrand Reinhold, New York

Shih, T. 1980. The evolution of coastlines and the development of tidal flats in western Taiwan (in Chinese). Geographical Research 6: 1-36.

Shuisky, Y.D. 1985a. Albania. pp. 443-444 in: E.C.F. Bird and M.L. Schwartz (editors), The World's Coastline. Van Nostrand Reinhold, New York.

Snead, R E. and M. Tasnif. 1966. Vegetation types in the Las Bela region of West Pakistan. Ecology 47: 494-499

Steers, J.A. 1960. Scolt Head Island. Cambridge University Press, Cambridge, 269 p

Swinbanks, D.D. and J.W. Murray. 1981. Biosedimentological zonation of Boundary Bay tidal flats, Fraser River Delta, British Columbia. Sedimentology 28: 201-237.

Thorn, B.G. 1984. Sand barriers of east Australia: Gippsland - a case study. pp. 233- 261 in: B.G. Thom (editor), Coastal Geomorphologi; in Australia. Academic Press, New York.

Thorn, B.G. 1985. New South Wales. pp. 969-974 in: E.CF. Bird and M.L. Schwartz (editors), The World's Coastline. Van Nostrand Reinhold, New York

Thom, B.G. and L.D. Wright. 1983. Geomorphology of the Purari Delta. pp. 47-65 in: T. Petr (editor), The Purari - Tropical Environment of a High Rainfall River Basin. Dr. Junk, Amsterdam.

Thompson, R.W. 1968. Tidal Flat Sedimentation on the Colorado River Delta, Northwestern Gulf of California. Geological Society of America. Memoir 107, 133 p. New York.

Thompson, R.W. 1975. Tidal-flat sediments of the Colorado River delta, northwestern Gulf of California. pp. 57-65 in: R.N. Ginsburg (editor), Tidal Deposits. Springer,

van Andel, T.H. 1967. The Orinoco delta. Journal of Sedimentary Petrology 37: 297-310.

Veenstra, H.J. 1980. Introduction to the geomorphology of the Wadden Sea. pp. 8-19 in: W.J. Wolf (editor), Geomorphology of the Wadden Sea Area. Balkema, Rotterdam

Von der Borch, C.C. 1976. Stratigraphy and formation of Holocene dolomitic carbonate deposits of the Coorong area, South Australia. Journal of Sedimentary Petrology 46: 952-966

Von der Borch, C.C., B. Bolton and J.K. Warren. 1977. Environmental setting and microstructure of subfossil lithified stromatolites associated with evaporites, Marion Lake, South Australia. Sedimentology 24: 693-708

Warren, J.K. 1982. The hydrological setting, occurrence and significance of gypsum in late Quaternary salt lakes in South Australia. Sedimentology 29: 609-637.

Warren, J.K. 1990. Sedimentology and mineralogy of dolomitic Coorong Lakes, South Australia. Journal of Sedimentary Petrology 60: 843-858.

Wells, J.T. and O.K. Huh. 1979. Tidal flat muds in the Republic of Korea: Chinhae to Inchon. Office of Naval Research Science Bulletin (Tokyo) 4: 21-30

Wolanski, E. and R.J. Gibbs. 1995. Flocculation of suspended sediment in the Fly River estuary, Papua New Guinea. Journal of Coastal Research 11: 754-762.

Wolanski, E., P. Ridd, B. King and M. Treorden. 1992. Fine sediment transport, Fly River estuary, Papua New Guinea. Report, OK Tedi Mining Ltd., Australian Institute of Marine Science, 31 p.

Woodroffe, C.D., R.J. Curtis and R.F. McLean. 1983. Development of a chenier plain, Firth of Thames, New Zealand. Marine Geology 53: 1-22.

Zhao, X. 1980. Holocene changes on the west coast of Bohai Bay. pp. 302-309 in: Academica Sinica (editor), The Origin and Development of the Fault-Block Region of Northern China. Science Press, Beijing.

Zhuang, W.-Y. and J. Chappell. 1991. Effects of seagrass beds on tidal flat sedimentation, Corner Inlet, southeast Australia. pp. 291-300 in: D.G. Smith, G.E. Reinson, B.A. Zaitlin and R.A. Rahmani (editors), Clastic Tidal Sedimentology. Canadian Society of Petroleum Geologists Memoir 16.

Zonneveld, J. 1954. Waarnemingen langs de kust van Suriname. Tijdschrift der Koninklijke Nederlands Aardrijkskundig Genootschap 71: 18-31.

Supplementary References

1. Alonso, A. C., Van Maren, D. S., Elias, E. P. L., Holthuijsen, S. J. & Wang, Z. B. The contribution of sand and mud to infilling of tidal basins in response to a closure dam. *Mar. Geol.* **439**, 106544 (2021).
2. Ploton, P. *et al.* Spatial validation reveals poor predictive performance of large-scale ecological mapping models. *Nat. Commun.* **11**, 4540, DOI: 10.1038/s41467-020-18321-y (2020).
3. Flemming, B. Geographic distribution of muddy coasts, in book: Muddy coasts of the world: Processes, deposits and function. (2002).
4. Salman, A., Lombardo, S. & Doody, P. Living with coastal erosion in europe: Sediment and space for sustainability. *Eurosion project reports* (2004).
5. Luijendijk, A. *et al.* The State of the World's Beaches. *Sci. Reports* **8**, 1–11, DOI: 10.1038/s41598-018-24630-6 (2018).
6. Alongi, D. M., da Silva, M., Wasson, R. J. & Wirasantosa, S. Sediment discharge and export of fluvial carbon and nutrients into the arafura and timor seas: A regional synthesis. *Mar. Geol.* **343**, 146–158, DOI: 10.1016/j.margeo.2013.07.004 (2013).
